# Acupuncture for nausea and vomiting during pregnancy: an overview of systematic reviews

**DOI:** 10.3389/fendo.2026.1886667

**Published:** 2026-08-07

**Authors:** Jingxuan Cui, Zhen Ai, Wenjuan Shen, Mei Han

**Affiliations:** 1 Heilongjiang University of Chinese Medicine, Heilongjiang, Harbin, China; 2 The First Affiliated Hospital of Heilongjiang University of Chinese Medicine, Harbin, China; 3 Centre for Evidence-Based Chinese Medicine, Beijing University of Chinese Medicine, Beijing, China

**Keywords:** acupuncture, meta-analysis, nausea, pregnancy, systematic review, vomiting

## Abstract

**Background:**

Nausea and vomiting during pregnancy (NVP) are common symptoms in early pregnancy. NVP can further affect the physical and mental health of pregnant women. Acupuncture has shown potential clinical value in treating NVP and can be considered a beneficial supplemental therapy for NVP. However, existing systematic reviews (SRs) and meta-analyses (MAs) on the efficacy and safety of acupuncture for NVP have yielded inconsistent conclusions, and their methodological and reporting quality has not yet been systematically and comprehensively evaluated.

**Objective:**

To synthesize the existing evidence on the efficacy and safety of acupuncture for the treatment of NVP and to assess the quality and risk of bias of the available SRs/MAs.

**Methods:**

Two researchers independently searched nine databases for relevant literature, screened studies according to predefined inclusion and exclusion criteria, and extracted data from the eligible SRs and MAs. The methodological quality, reporting completeness, risk of bias, and strength of evidence were rigorously evaluated using AMSTAR 2, PRISMA-A, ROBIS, and GRADE, respectively. In addition, the GROOVE tool was used to assess the degree of overlap among the original studies by calculating the corrected covered area (CCA).

**Results:**

We retrieved a total of 288 studies, and 9 SRs/MAs were ultimately included in this review. The methodological quality of one study was moderate, while two studies were rated as low and the remaining six as critically low. According to the PRISMA-A statement, the reporting items of the included SRs/MAs were relatively complete. Furthermore, it revealed a slight overlap among the original studies, with a CCA of 3.64%. The results based on GRADE showed that of the 52 outcomes, 2 were rated as moderate quality, 20 as low quality, and 30 as very low quality. Descriptive analysis showed that acupuncture combined with medication could reduce the frequency of nausea and vomiting, accelerate the ketonuria-negative rates, and maintain serum electrolyte balance. Acupuncture also appeared to reduce the frequency of nausea and vomiting compared with medication alone.

**Conclusions:**

Acupuncture appears to relieve the symptoms of NVP to a certain extent. However, the current evidence is limited by low reliability, methodological flaws, publication bias, and inaccurate data. Therefore, the true efficacy remains unclear. Although acupuncture shows preliminary clinical potential, high-quality randomized controlled trials with standardized outcome indicators are still needed to draw definite conclusions.

**Systematic review registration:**

https://www.crd.york.ac.uk/PROSPERO/, identifier CRD420261326889.

## 1 Introduction

Nausea and vomiting during pregnancy (NVP) refers to nausea and vomiting occurring before 16 weeks of gestation in the absence of other identifiable causes, and it is one of the most common symptoms of early pregnancy (1, 2). Previous studies have shown that approximately 50%–80% of pregnant women experience varying degrees of NVP during early pregnancy (3). Persistently symptomatic NVP may lead to numerous adverse outcomes, including marked ketonuria, excessive weight loss, malnutrition, dehydration, and electrolyte imbalances (4, 5). High levels of ketonuria can exceed the metabolic load on the kidneys, thereby affecting pregnancy outcomes and threatening maternal life (6, 7). This severe form of pregnancy-related vomiting is known as hyperemesis gravidarum (HG) (8, 9). In addition, approximately 10% of women with HG terminate their pregnancies because of the severity of their symptoms (10). Both NVP and HG impair daily life and impose a substantial mental burden on pregnant women (11). Studies have also shown that the economic burden of NVP on families and society should not be underestimated (12, 13). Consequently, NVP is an important healthcare issue that deserves greater attention.

Currently, there is still no ideal treatment for NVP in clinical practice, and existing interventions mainly focus on symptom relief and the maintenance of fluid and electrolyte balance (14, 15). For patients with HG, the primary goals of clinical management are to rapidly control vomiting and correct dehydration and malnutrition, thereby ensuring maternal safety and normal fetal development (16, 17). In Western medicine, symptomatic supportive treatments, such as oral medications, antiemetic therapy, and fluid replacement, are commonly used (18, 19). Although these interventions improve symptoms in some patients, others still choose to terminate their pregnancies because of unsatisfactory efficacy or intolerance to treatment (20). In addition, the use of some antiemetic drugs is restricted because of concerns about potential teratogenicity (21–23). Survey data have also shown that some patients with HG are unable to eat or take medications because of severe nausea and vomiting, which may negatively affect treatment outcomes (24, 25). Therefore, safe and effective therapeutic options for NVP are still needed.

Acupuncture is an important component of traditional Chinese medicine therapies (26, 27). By providing beneficial stimulation to specific acupoints, it is believed to promote the transmission of meridian qi along the channels, thereby regulating the functions of the zang-fu organs (28). Acupuncture has several advantages, including good therapeutic effects, a high safety profile, and convenient application (29). Its main modalities include manual acupuncture, moxibustion, acupoint injection (AIT), press-needle therapy, herbal acupoint application (AAT), auricular acupressure (APA), and combination therapies (30–33). Acupuncture has been shown to have beneficial therapeutic effects in a variety of gynecological disorders and is generally considered safe (34).

In recent years, acupuncture has been increasingly used in the clinical management of NVP (32, 33). Acupuncture treatment for NVP is typically individualized according to symptom severity, patient characteristics, and gestational age (35). Previous studies have suggested that acupuncture may offer important clinical benefits for the treatment of NVP (36). Commonly selected acupoints include Shangwan (CV13), Zhongwan (CV12), Xiawan (CV10), Zusanli (ST36), and Neiguan (PC6), which are used to relieve nausea and vomiting, in addition to Zhongfeng (LR4), Taichong (LR3), Baihui (DU20), and Sishencong (EX-HN1), which may help regulate bodily functions and improve emotional wellbeing (37). Yan (38) reported that, compared with the control group, patients receiving acupuncture at these acupoints had a shorter time to urinary ketone normalization, faster improvement in vomiting symptoms, and higher patient satisfaction. Therefore, further evidence synthesis is still needed to comprehensively evaluate the efficacy and safety of acupuncture for NVP.

Systematic reviews (SRs) and meta-analyses (MAs) represent the highest level of evidence in evidence-based medicine and provide important support for clinical decision-making (39). However, SRs/MAs on the same topic often vary in quality and may yield conflicting conclusions, thereby limiting their value in guiding clinical practice. An overview of systematic reviews is a secondary evidence synthesis approach that can comprehensively evaluate the reporting quality, methodological quality, and overall risk of bias of relevant SRs/MAs, while also grading the certainty of evidence for their reported outcomes (40, 41). Therefore, this study conducted an overview to comprehensively assess the quality of the included reviews, thereby providing stronger evidence-based support for the rational clinical use of acupuncture for NVP.

## 2 Methods

### 2.1 Protocol and registration

The protocol for this overview was registered with the International Prospective Register of Systematic Reviews (PROSPERO) (registration No. CRD420261326889). There was no requirement for ethics approval.

### 2.2 Literature search

The researchers searched nine electronic databases: PubMed, Embase, the Cochrane Library, Web of Science, CINAHL, Chinese Biological Medicine, China National Knowledge Infrastructure, Wanfang, and the Chinese Science and Technology Journals Databases. In addition, relevant registered SRs on the PROSPERO platform were searched manually, and the reference lists of included studies were screened to identify potentially eligible publications as comprehensively as possible. The literature search used Medical Subject Headings (MeSH) and free-text terms, such as “acupuncture,” “nausea and vomiting during pregnancy,” “systematic review,” and “meta-analysis.” The search strategy was adapted and optimized according to the search requirements and characteristics of each database. The search period covered the period from database inception to 5 March 2026. The search was limited to publications in Chinese and English. Detailed search strategies are provided in the Appendix.

### 2.3 Eligibility criteria

#### 2.3.1 Inclusion criteria

SRs/MAs were included if they met the following criteria:

1. Study design: SRs/MAs that included randomized controlled trials (RCTs).
2. Participants: No diagnostic criteria were used because NVP was judged as symptomatic. Eligible participants were pregnant women who reported currently experiencing pregnancy-related nausea and vomiting.
3. Interventions: Acupuncture was used as the primary treatment or as an important additional treatment, including manual acupuncture, moxibustion, electroacupuncture, warm acupuncture, abdominal acupuncture, auricular acupuncture, catgut embedding at acupoints, and acupoint application.
4. Control: Sham acupuncture (SA), placebo, no treatment, usual care, medications, or intravenous fluid therapy (ivgtt).
5. Type of outcomes: Reduction or cessation of NVP symptoms evaluated using different scales, such as the Pregnancy-Unique Quantification of Emesis (PUQE), the Rhodes Index of Nausea, Vomiting and Retching Scale, and ineffective rates; ketonuria; length of stay (LOS); NVP Quality of Life (NVPQOL); psychological disorders, such as anxiety and depression; Chinese Medicine Syndrome Scale (CMS) score; recurrence rate; pregnancy termination rate; and adverse events (AEs).

#### 2.3.2 Exclusion criteria

SRs/MAs were excluded if they met any of the following criteria:

1. They were not SRs/MAs.
2. The participants were diagnosed with NVP combined with other diseases.
3. Acupuncture was not the sole difference between the intervention and control groups.
4. The full text was not available for review.

### 2.4 Study selection and data extraction

#### 2.4.1 Study selection

All retrieved records were imported into EndNote X9 (Thomson ResearchSoft, New York, NY, USA) for centralized management. After removing duplicates, two reviewers (CJX and AZ) independently screened the titles and abstracts to identify records that were potentially eligible, followed by cross-checking their screening results. Any disagreements regarding study eligibility were resolved through discussion. If consensus could not be reached, a third reviewer (SWJ) was consulted, and the final decision was made accordingly.

#### 2.4.2 Data extraction

Two reviewers (CJX and AZ) independently extracted data from the included SRs/MAs using a standardized, predefined data extraction form. The extracted information included the first author, year of publication, number of included RCTs and total sample size, intervention details for both treatment and control groups, primary and secondary outcomes, methodological assessment tools, data analysis methods, and study conclusions. For duplicate publications, data extraction and quality assessment were performed after consolidating the relevant information. Any discrepancies in the extracted data were first resolved through discussion between the two reviewers. If disagreement persisted, a third reviewer (SWJ) adjudicated to ensure the accuracy and consistency of the extracted data.

### 2.5 Assessment methods

The study primarily followed the Cochrane Handbook and associated methodologies for highly systematic evaluations and re-evaluations. The evaluation of quality comprised four dimensions: methodological quality, reporting quality, evidence quality, and risk of bias. Two investigators (CJX and AZ) independently carried out the assessment. Differences between how these reviewers scored the individual manuscripts were settled through discussion or consultation with the third reviewer (SWJ).

#### 2.5.1 Methodological quality assessment

The methodological quality of the included SRs/MAs was evaluated using the AMSTAR 2 tool. AMSTAR 2 is a validated tool for assessing SRs/MAs, consisting of 16 items, including 7 critical items and 9 non-critical items. Items 2, 4, 7, 9, 11, 13, and 15 are considered critical domains. The judging criteria for methodological quality were as follows: high, moderate, low, and critically low (42). Specifically, a review was rated as moderate if it had no more than one non-critical weakness; low if it had one critical flaw; and critically low if it had more than one critical flaw, regardless of whether the non-critical items were satisfied.

#### 2.5.2 Risk of bias assessment

The ROBIS tool was used to assess the risk of bias in the included reviews. The assessment process primarily focused on Phases 2 and 3 to determine different levels of risk of bias. Phase 2 involves identifying concerns with the review process across four domains: study eligibility criteria, identification and selection of studies, data collection and study appraisal, and synthesis and findings. Phase 3 provides an overall judgment of risk of bias based on the assessments made in each domain. The risk of bias for each domain was judged as low, high, or unclear (43).

#### 2.5.3 Reporting quality assessment

PRISMA-A is an extension of the PRISMA statement developed specifically for assessing the reporting quality of acupuncture-related SRs/MAs. This checklist consists of 27 items and was used to evaluate both individual items and the overall completeness of reporting in the included reviews. Each item was rated as yes, no, or partial yes (44). A quantitative scoring method was applied based on the completeness of reporting for each item and the overall reporting status of each review. A score of 1 was assigned when an item was fully reported, 0.5 when it was partially reported, and 0 when it was not reported.

#### 2.5.4 Certainty of evidence assessment

The quality of evidence was assessed using the Grading of Recommendations Assessment, Development and Evaluation (GRADE) approach (45, 46). Under this framework, evidence certainty was categorized into four levels (high, moderate, low, and very low) based on five downgrading factors: limitations, inconsistency, indirectness, imprecision, and publication bias.

#### 2.5.5 Overlap calculation

Data are presented as values, with descriptive analyses based on different analytical outputs. The GROOVE tool was adopted to generate citation overlap matrices and calculate the corrected covered area (CCA) (47). CCA values were calculated as follows: CCA (%) = (N - r)/(rc - r), where N represents the total number of included publications, r represents the number of rows (RCTs), and c represents the number of columns (SRs). CCA values were interpreted as follows: 0%–5% indicates mild overlap, 6%–10% moderate overlap, 11%–15% high overlap, and values greater than 15% very high overlap.

### 2.6 Data synthesis

We conducted a descriptive analysis of the included SRs/MAs. The item-level assessment results generated using AMSTAR 2, ROBIS, PRISMA-A, and GRADE were summarized and presented as frequencies and percentages. The corrected covered area (CCA) was calculated to assess overlap among the original studies. Data from the included reviews were synthesized with a summary of meta-analysis or a narrative description.

## 3 Results

### 3.1 Literature search and selection

**Figure 1:**
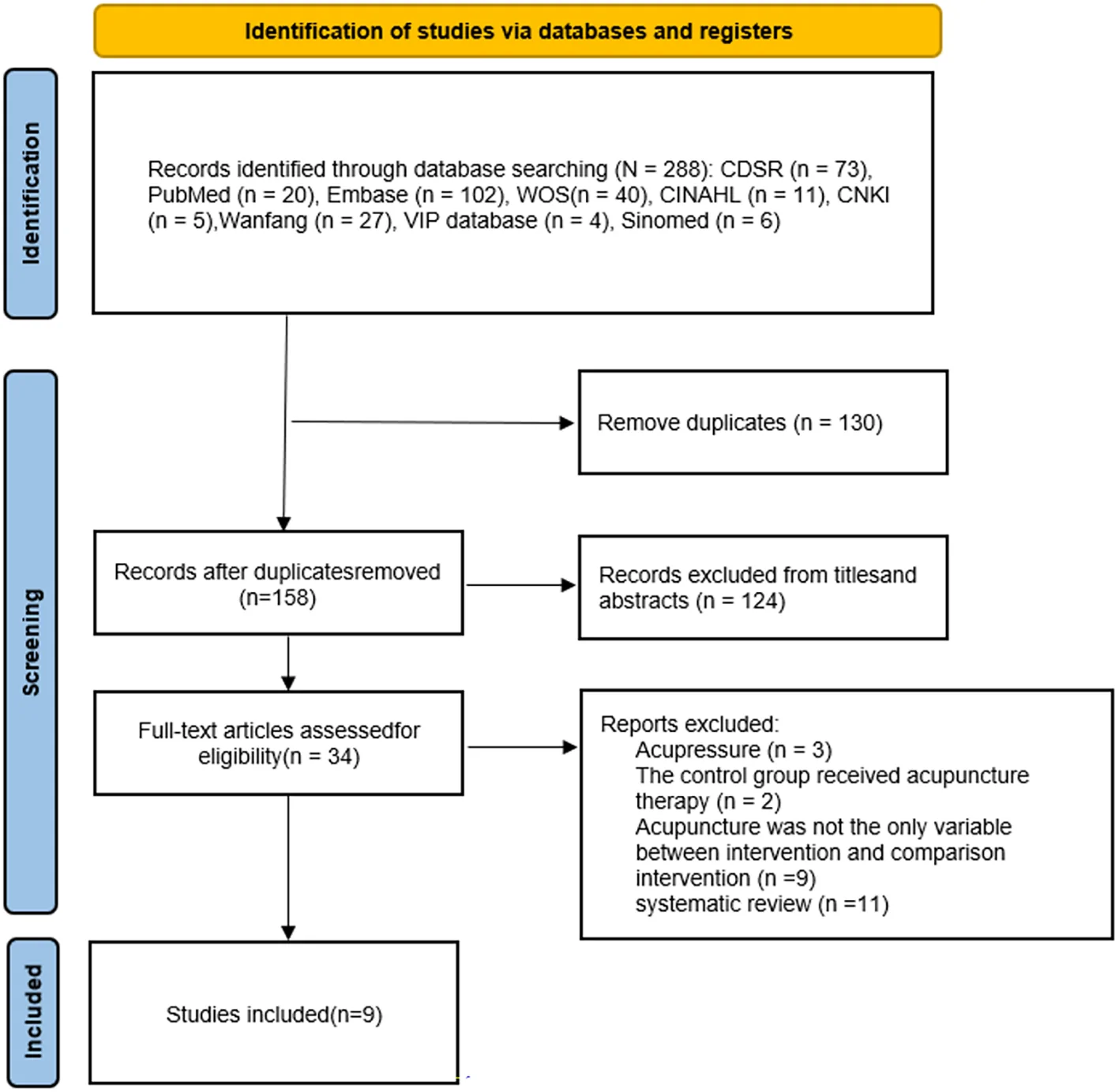
Flow diagram of the study selection process.

Following the pre-formulated search strategy, 288 original studies were initially identified. After removing duplicates (n = 130), 124 articles were excluded by screening their titles and abstracts, and the full texts of the remaining 34 articles were reviewed. After full-text assessment, 9 studies were included. The study selection process is shown in **Figure 1**.

### 3.2 Characteristics of the included SRs/MAs

The study included nine SRs/MAs, all of which focused on patients with NVP. Because the clinical diagnosis of NVP is largely based on symptom presentation, none of the included reviews applied uniform diagnostic criteria. The number of RCTs included in the nine SRs/MAs ranged from 5 to 24, with sample sizes ranging from 834 to 2,672. Eight SRs/MAs conducted MAs. Regarding interventions, acupuncture was the main treatment in the experimental groups and was delivered in four main forms: acupuncture alone, acupuncture combined with medication, acupuncture combined with other acupuncture-related therapies, and acupuncture combined with conventional treatment. Specific acupuncture methods included manual acupuncture, moxibustion, electroacupuncture, abdominal acupuncture, auricular acupuncture, catgut embedding at acupoints, acupoint injection, and acupoint application. Control interventions included sham acupuncture, placebo, no treatment, and usual care.

The reported outcomes were divided into eight groups: efficacy outcomes (effective rates, cure rates, and ineffective rates); improvement in nausea and vomiting (Pregnancy-Unique Quantification of Emesis [PUQE], frequency of nausea and vomiting, and improvement rate of nausea and vomiting symptoms); laboratory parameters (urinary ketones and serum potassium); quality of life and functional recovery outcomes (NVP Quality of Life [NVPQOL], length of stay (LOS), improvement in dietary intake/food consumption, Chinese Medicine Syndrome Scale (CMS) score, and improvement in fatigue symptoms); incidence of adverse events; recurrence rate; pregnancy termination rate; and psychological problems (anxiety and depression scores). **Supplementary Table 1** provides basic characteristics of the included systematic reviews and meta-analyses.

### 3.3 Results of quality assessment

#### 3.3.1 Methodological quality assessment

**Figure 2:**
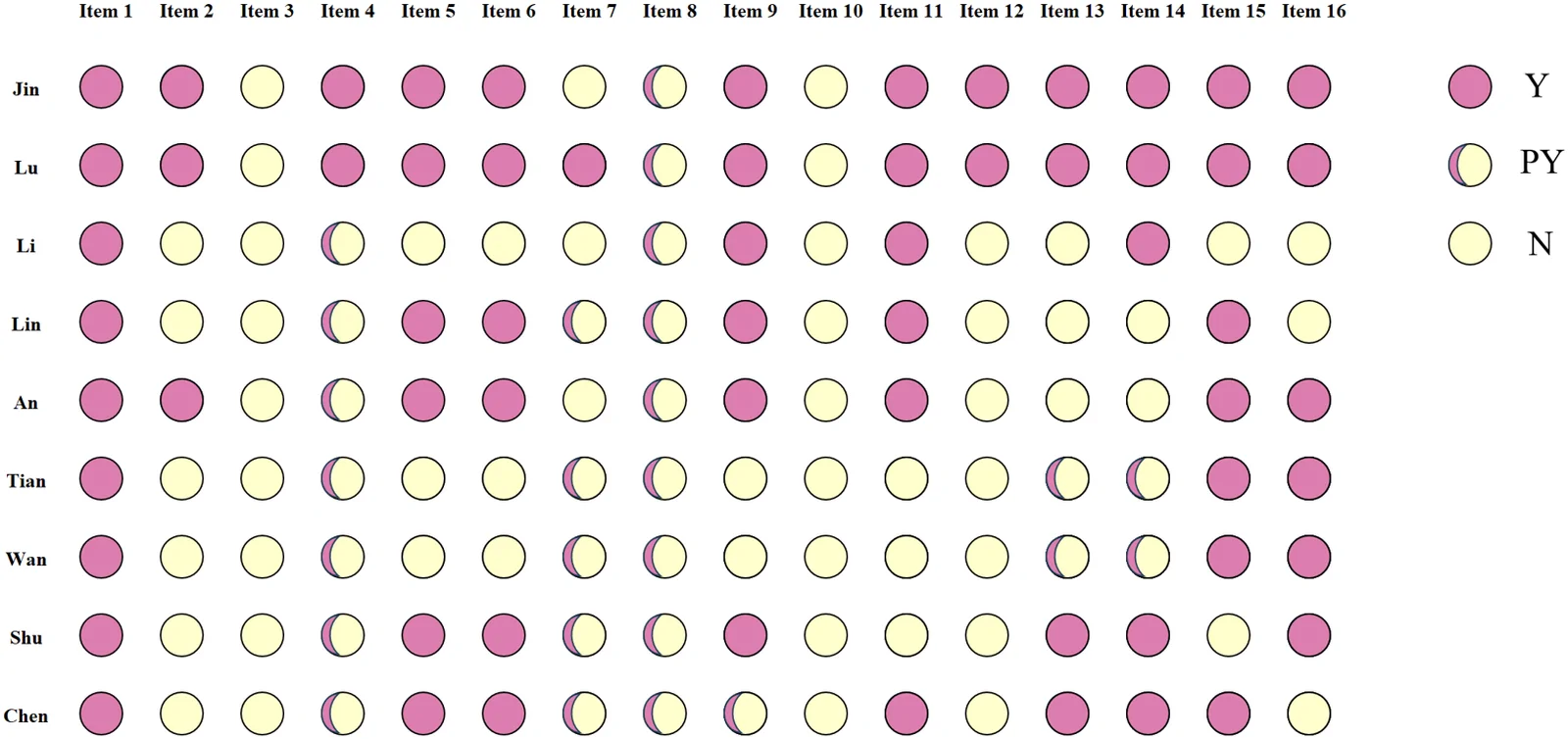
Evidence map of the methodological quality.

AMSTAR 2 indicated that the methodological quality of one SR was moderate, while two had low quality and six had critically low quality. For the critical items, the numbers and proportions of reviews rated as fully compliant (“Yes”) were as follows: Item 2, three (33.33%); Item 4, two (22.22%); Item 7, one (11.11%); Item 9, six (66.67%); Item 11, six (66.67%); Item 13, four (44.44%); and Item 15, seven (77.78%). Overall, the reporting of the non-critical items was suboptimal. However, Item 1 was fully reported in all included reviews (100%). For the remaining non-critical items, the compliance rates were as follows: Item 1, nine (100%); Item 3, zero (0%); Item 5, six (55.56%); Item 6, six (66.67%); Item 8, zero (0%); Item 10, zero (0%); Item 12, two (22.22%); Item 14, five (55.56%); and Item 16, six (66.67%). Varying degrees of deficiencies were identified across these items. Detailed AMSTAR 2 assessment results are presented in **Supplementary Table 2**, **Figure 2**.

#### 3.3.2 Risk of bias assessment

The ROBIS assessment results are presented in **Supplementary Table 3**. In Phase 1, all SRs/MAs were assessed as having a low risk of bias. However, in Phase 2, four SRs/MAs were considered low risk in Domain 1, which evaluated study eligibility criteria. Domain 2 covered the identification and selection of studies, for which six reviews were rated as high risk. For Domain 3, which evaluated data collection and study appraisal, six SRs/MAs were rated as low risk, and one SR was rated as having an unclear risk of bias. Five SRs/MAs had a low risk of bias in Domain 4, which examined the synthesis and findings. As for Phase 3, three SRs/MAs were judged to have an overall low risk of bias.

#### 3.3.3 Reporting quality assessment

**Figure 3:**
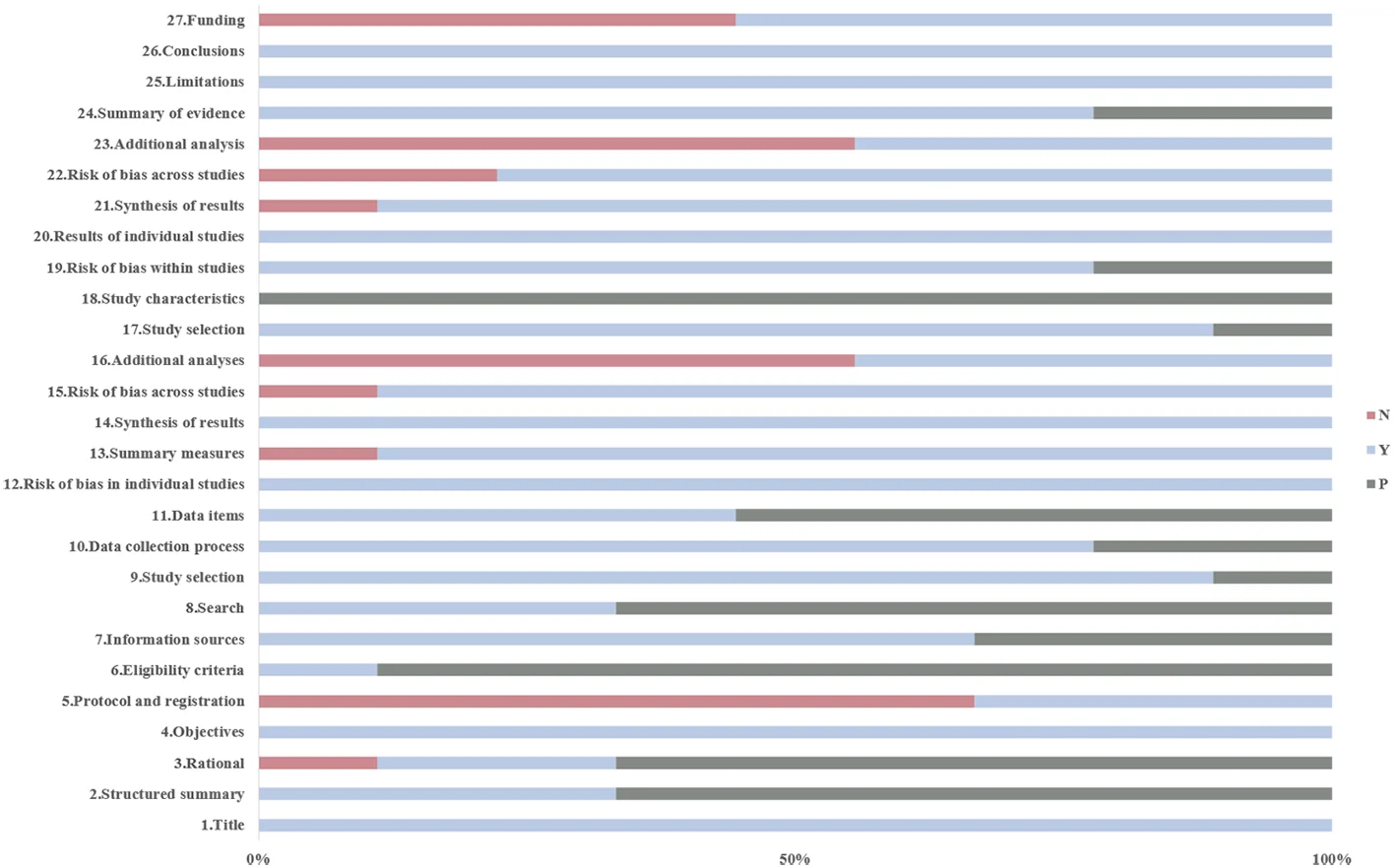
Reporting quality assessment by PRISMA-A.

Of the 27 items, 23 had a “yes” or “partially yes” response rate of more than 70%, indicating relatively complete reporting. However, reporting deficiencies were found in some items. The results showed that the overall reporting quality was not optimal. There were four substantial deficiencies in reporting: Item 5, Item 16, Item 23, and Item 27. Regarding study registration, only three SRs/MAs prospectively registered their study protocols. Three SRs/MAs did not report funding information, whereas four SRs/MAs provided detailed funding statements, including two that explicitly stated that no funding was received. The detailed reporting results are presented in **Supplementary Table 4**, **Figure 3**.

**Figure 4:**
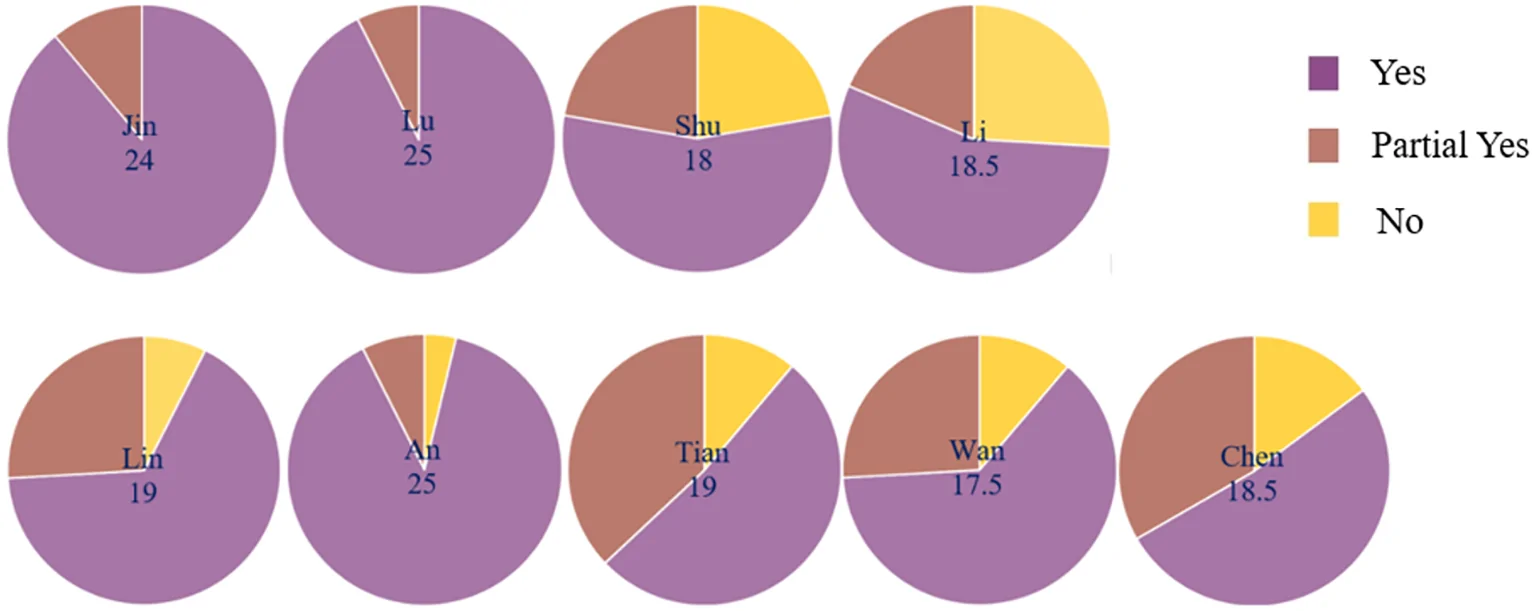
Overall reporting quality assessment of each study included in the systematic review.

Across the nine SRs/MAs, the overall reporting completeness scores ranged from 17.5 to 25.0. Reviews (48, 49) had the fewest reporting deficiencies, whereas review (50) had the greatest number of missing items. The overall reporting quality of each review is presented in **Figure 4**.

#### 3.3.4 Certainty of evidence assessment

We summarized the confidence in the findings of nine SRs/MAs in **Supplementary Tables 5–7**. According to the GRADE assessment, 2 (3.84%) were rated as having moderate quality, 20 (38.46%) as low quality, and 30 (57.69%) as very low quality. Among the factors leading to downgrading, risk of bias (100%) and publication bias (96.15%) were the most common.

##### 3.3.4.1 Effects of acupuncture on nausea and vomiting

###### 3.3.4.1.1 PUQE score

Three SRs/MAs (49, 51, 52) reported no significant difference between the acupuncture group and the control group in reducing PUQE scores, and the certainty of evidence was very low (MD = –0.80, 95% CI: –3.06 to 1.46). One SR (52) reported that acupuncture improved nausea and vomiting symptoms compared with medications, although the certainty of evidence was very low (MD = –2.67, 95% CI: –4.25 to –1.08). Compared with usual care, one SR (49) found that various acupuncture-related interventions, including acupoint application, manual acupuncture, intradermal needling, and auricular acupressure, when combined with usual care, improved nausea and vomiting symptoms; the quality of evidence was low (P < 0.001). One SR (51) reported no significant difference between auricular acupressure alone and SA, with very low-quality evidence (P < 0.001).

###### 3.3.4.1.2 Improvement rate of nausea and vomiting symptoms

One SR (53) with low-quality evidence compared the efficacy of acupuncture plus ivgtt against ivgtt alone, and the results showed that acupuncture plus ivgtt was more effective in improving the rate of symptom relief for nausea and vomiting (OR = 26.44, 95% CI: 3.54 to 197.31). Another SR (48) with low-quality evidence showed that acupuncture was superior to other therapies for improving nausea and vomiting symptoms, while another SR (49) found no difference between the two groups (P < 0.001).

##### 3.3.4.2 Effects of acupuncture on laboratory indicators

###### 3.3.4.2.1 Ketonuria-negative rates

Three SRs/MAs with very low-certainty evidence (48, 52, 54) showed no difference between acupuncture and medications in promoting ketonuria-negative rates (RR = 1.32, 95% CI: 1.14 to 1.53; RR = 1.27, 95% CI: 0.85 to 1.90; RR = 1.36, 95% CI: 1.15 to 1.60; OR = 4.85, 95% CI: 0.86 to 27.22). Two SRs/MAs (53, 55) compared APA or AAT against ivgtt, and both studies found that APA or AAT was more effective than the control in improving ketonuria-negative rates (OR = 7,51, 95% CI: 4.05 to 13.90; OR = 3.78, 95% CI: 1.86 to 7.67).

###### 3.3.4.2.2 Serum potassium

In addition, regarding the comparative efficacy of acupuncture combined with medications, one SR (48) found that acupuncture plus medications was significantly more effective than medications alone in improving serum potassium levels, although the quality of evidence was very low (MD = 0.11, 95% CI: –0.04 to 0.25). Another SR with low-quality evidence (54) reported that acupuncture plus ivgtt was more effective than ivgtt (MD = 0.11, 95% CI: –0.04 to 0.25).

##### 3.3.4.3 Effects of acupuncture on quality of life and functional recovery

###### 3.3.4.3.1 NVPQOL

One SR (52) with low-quality evidence showed that acupuncture was superior to medications in reducing NVPQOL scores (MD = –10.89, 95% CI: –13.92 to 7.86).

###### 3.3.4.3.2 LOS

Three SRs/MAs (48, 52, 54) evaluated the efficacy of acupuncture compared with other therapies in shortening the length of hospital stay (LOS). Among them, one SR (52) compared acupuncture plus medications with medications alone and found that the combination was more effective (SMD = –2,32, 95% CI: –3.01 to 1.63). One study (48) compared acupuncture with medications and found that acupuncture was more effective (MD = –3.78, 95% CI: –5.39 to –2.16). Another SR (54) reported that acupuncture was significantly superior to ivgtt in reducing LOS (MD = –2.89, 95% CI: –4.01 to –1.77).

###### 3.3.4.3.3 Improvement in dietary intake/food consumption

One SR (53) with low-quality evidence compared the efficacy of acupuncture plus medications against medications alone, and the results showed that acupuncture plus medications was more effective than medications alone in improving dietary intake (MD = 0.11, 95% CI: –0.04 to 0.25).

###### 3.3.4.3.4 CMS

One SR (52) with low-quality evidence evaluated acupuncture plus medications versus medications for improving CMS, and the results demonstrated that acupuncture plus medications resulted in a higher CMS score (SMD = –0.67, 95% CI: –0.99 to –0.36).

###### 3.3.4.3.5 Improvement in anxiety and depression

One SR with low-quality evidence (52) showed that, compared with medications, acupuncture plus medications or acupuncture alone both showed a better reduction effect on improving anxiety and depression scores.

###### 3.3.4.3.6 Improvement in fatigue

One study with very low-quality evidence (48) showed that acupuncture plus medications was better than usual care alone for improving fatigue (RR = 1.01, 95% CI: 0.89 to 1.14).

##### 3.3.4.4 Overall effectiveness

###### 3.3.4.4.1 Overall response rate

Three SRs/MAs (49, 53, 55) reported that acupuncture improved the overall clinical response rate when compared with control; of these studies, two (49, 50) had low evidence quality and one (53) had moderate evidence quality. Two SRs/MAs with low-quality evidence (51) and very low-quality evidence (56) showed that, compared with control, APA improved the overall clinical response rate. A study with very low-quality evidence (49) showed that acupuncture-related therapies were better than mixed usual care.

One SR (49) compared acupuncture plus usual care with usual care alone, and the results showed that acupuncture plus usual care was more effective in improving the overall clinical response rate. Three SRs/MAs with low-certainty evidence (48, 54, 55) reported that acupuncture plus control was more effective than control alone.

###### 3.3.4.4.2 Cure rate

A study with moderate-quality evidence (55) showed that, compared with ivgtt, AIT showed a better effect on improving cure rate (OR = 4.18, 95% CI: 3.02 to 5.78). Two SRs/MAs (50, 56) with low-quality evidence suggested that, when compared with ivgtt, AIT and AAT were slightly better for improving cure rates (OR = 3.25, 95% CI: 2.93 to 4.52; OR = 2.42, 95% CI: 2.14 to 5.45).

###### 3.3.4.4.3 Ineffective rates

A study with very low-quality evidence (52) showed that acupuncture was better than medications for reducing ineffective rates (RR = 0.50, 95% CI: 0.30 to 0.81).

##### 3.3.4.5 Recurrence rate

A study with low-quality evidence (53) suggested that AAT plus ivgtt had a better effect in reducing the risk of recurrence, compared with ivgtt alone (RR = 0.14, 95% CI: 0.08 to 0.24).

##### 3.3.4.6 Pregnancy termination rate

Two SRs/MAs with low-quality evidence (48, 54) reported that, compared with ivgtt or medications, acupuncture reduced the pregnancy termination rate (OR = 0.14, 95% CI: 0.02 to 0.75; RR = 0.26, 95% CI: 0.09 to 0.74).

##### 3.3.4.7 Adverse events

One SR (48) found that the incidence of adverse events was significantly lower in the acupuncture group compared with the ivgtt group (RR = 0.13, P = 0.04). Another SR (52) comparing acupuncture with medications found no statistical difference in serious adverse events (RR = 0.67, P = 0.57). No significant difference in serious adverse reactions existed when acupuncture was compared with sham acupuncture (RR = 0.64, P = 0.29). The remaining SR (53) was not quantitatively synthesized, with only one RCT documenting mild adverse reactions.

#### 3.3.5 Overlap of primary studies

**Figure 5:**
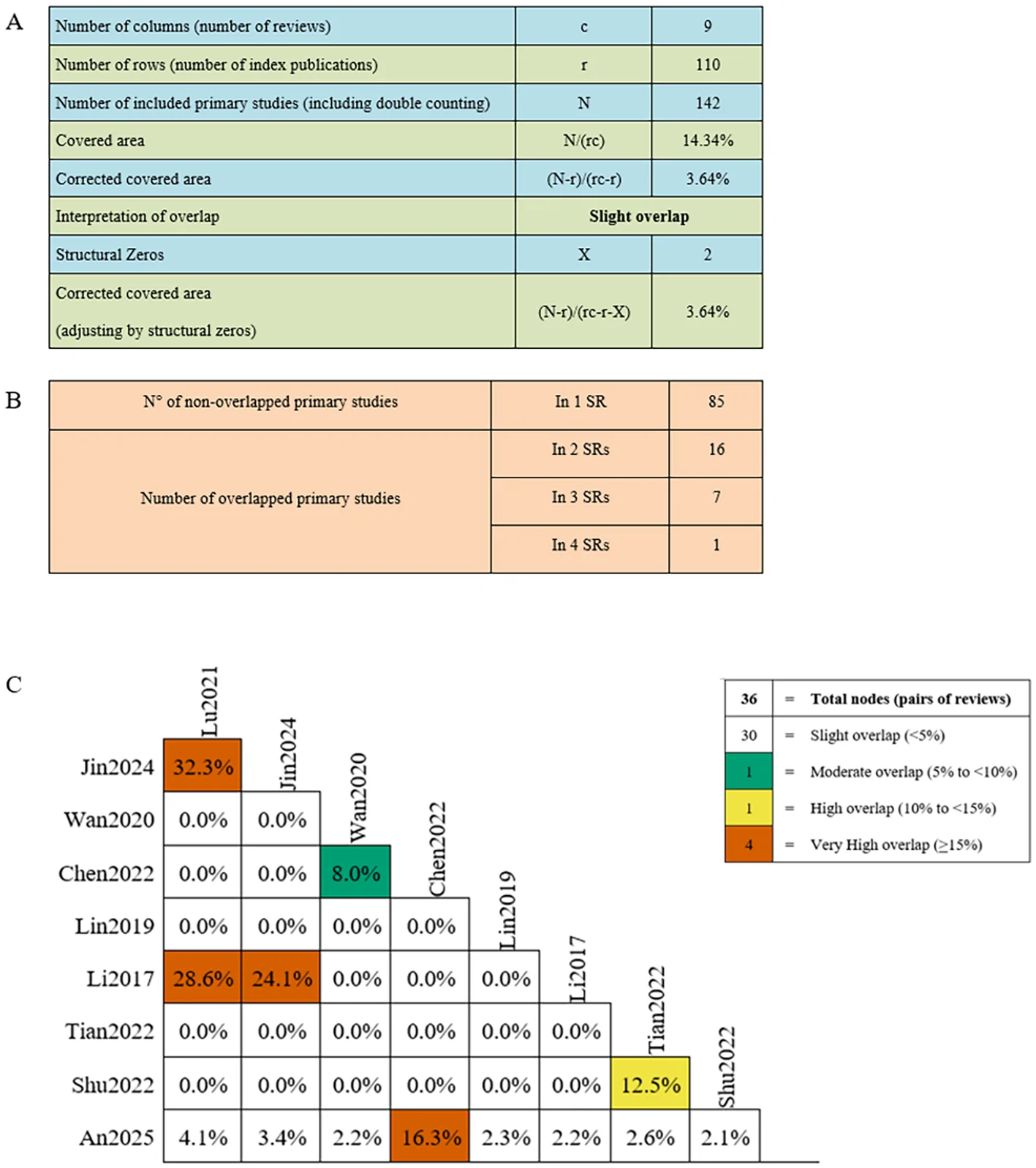
Matrix diagram of GROOVE. **(A)** Overall overlap results using the GROOVE tool; **(B)** Number of non-overlapped and overlapped primary studies; **(C)** Graphical representation of overlap for overviews.

We used the GROOVE tool to calculate the citation overlap across the nine SRs/MAs. The overall overlap rate reached 3.64%. This value suggested minimal overlap among included studies (**Figure 5A**). A total of 85 unique studies were identified from these nine SRs/MAs. A total of 16 studies appeared in two SRs, 7 studies appeared in three SRs, and 1 study was cited by four SRs (**Figure 5B**). Each node in **Figure 5C** reflects the overlap between two SRs. Among all 36 nodes (boxes), 30 nodes showed mild overlap. In addition, 1 node showed moderate overlap, and another showed high overlap. Only 4 nodes showed very high overlap.

## 4 Discussion

This overview integrated and summarized the existing SRs/MAs on acupuncture for NVP. The aim was to evaluate the evidence base regarding the safety and efficacy of acupuncture for NVP. By assessing the quality of the existing SRs/MAs and the level of evidence, we identified the following main findings.

### 4.1 Main findings

Our results show that acupuncture can significantly relieve clinical symptoms and improve multiple NVP-related functional indicators, including PUQE, Rhodes Index, NVPQOL, and CMS. This therapy also improves objective clinical outcomes such as ketonuria levels and length of stay (LOS). However, several SRs/MAs still demonstrated important methodological shortcomings. Evidence rated as high or moderate quality was considered relatively reliable. This relatively reliable evidence suggests that acupuncture alone can improve nausea and vomiting symptoms in patients with NVP. Compared with ivgtt alone, acupuncture plus usual care may be more effective in improving ketonuria outcomes. Compared with usual care alone, acupuncture plus usual care may be more effective in improving ketonuria outcomes. In terms of safety, current evidence remains limited. Only two SRs conducted quantitative analyses of adverse events, while most studies failed to systematically assess adverse events. Overall, available data on the safety of acupuncture are insufficient and poorly reported. No severe adverse events have been documented; however, existing evidence cannot confirm the safety of acupuncture therapy. Future studies should record adverse events in a stricter and more standardized manner. Regarding the effects of acupuncture on PUQE, anxiety, depression, fatigue, serum potassium, NVPQOL, LOS, dietary intake, CMS, and related outcomes, the confidence in current evidence was too low to draw conclusions.

Our evaluation of the methodological quality of the nine included SRs/MAs using AMSTAR 2 revealed concerning limitations. Only one SR achieved a moderate-quality rating, while two were rated as low quality and six as critically low quality. Several key methodological shortcomings were identified: most reviews lacked pre-registered research protocols, failed to document their exclusion criteria in detail, omitted funding source information, and inadequately disclosed potential conflicts of interest. Although many reviews employed appropriate search strategies and statistical methods, these fundamental limitations affected the reliability of their findings. Similarly, the ROBIS analysis in individual SRs/MAs was not adequately explained or discussed during the interpretation of the results. This may have limited the ability to interpret the reliability of the results. The PRISMA-A evaluation showed that in the vast majority of SRs/MAs, pre-registered or pre-published study protocols, complete search strategies, detailed literature screening processes, necessary additional analyses, and clear funding information were not adequately reported, which affected the transparency and reporting quality. Under GRADE, the most common reasons for downgrading were publication bias, followed by imprecision.

### 4.2 Implications for future research

Our review has several implications for future research. Further higher-quality SRs/MAs are necessary to provide solid evidence regarding acupuncture for NVP. We strongly suggest that future reviewers register the protocols before initiating their reviews. Given the generally low quality of the current evidence, well-designed, high-quality RCTs on acupuncture for NVP, particularly in patients with HG, are warranted to determine its beneficial effects. Meanwhile, according to the Preferred Reporting Items for Systematic reviews and Meta-Analyses (PRISMA-A), authors should register their protocols before conducting systematic reviews, provide a list of excluded studies if possible, and report their reviews according to established criteria. Importantly, as reported in the majority of published SRs, the original clinical trials were poorly conducted; therefore, efforts to systematically review these RCTs should first focus on improving the quality of the underlying clinical trials. Finally, the number of eligible studies for each monotherapy is insufficient to conduct separate subgroup analyses. In addition, multiple acupoint stimulation modalities inevitably lead to high heterogeneity. Future trials should recruit adequate sample sizes to enable subgroup analyses focusing on individual intervention types.

### 4.3 Strengths and weaknesses of the review

Our study employed AMSTAR 2 and ROBIS to assess the methodological quality and risk of bias of reviews evaluating the effectiveness of acupuncture for NVP. We determined the strength of the evidence using the GRADE approach. The overview protocol was developed in advance and registered in PROSPERO, which helped limit the possibility of biased decisions being made during the review process. In addition, the degree of overlap among primary studies was quantified using the CCA via the GROOVE tool. The overall CCA was 3.64%, indicating a slight overlap among included reviews. This low level of redundancy indicates that the included SRs and MAs were largely independent, thereby minimizing the risk of inflated or biased conclusions resulting from duplicated primary studies.

However, this overview faces important limitations that warrant careful consideration. The majority of the included RCTs had relatively small sample sizes and demonstrated concerning methodological weaknesses that could introduce bias. The lack of standardization in acupuncture protocols, including acupoint prescriptions and manipulation techniques, makes it difficult to describe and evaluate the interventions in depth; this also limits the ability to draw firm conclusions regarding effectiveness. Furthermore, none of the included studies performed detailed statistical analyses of the effective rate. No confounding factors were adjusted for this outcome, which may have impaired the reliability of our findings to some extent. In addition, although the exclusion of several ineligible randomized controlled trials improved interstudy homogeneity, it reduced the total sample size and introduced selection bias, which may compromise the accuracy of effect estimates and the generalizability of the results. Lastly, the following limitations of this study should be considered: (1) language bias, as only studies published in Chinese and English were included; (2) potential publication bias resulting from the exclusion of grey literature; and (3) heterogeneity induced by pooling diverse acupuncture modalities, which may have influenced the pooled results.

## 5 Conclusion

Current evidence indicates that acupuncture, as an important adjunctive therapy for NVP, can improve nausea and vomiting symptoms, CMS, ketonuria, serum potassium, and quality of life. However, the poor quality and inconsistency of the existing evidence make it difficult to determine whether acupuncture is effective and safe for NVP based on the current evidence. Therefore, further high-quality studies are needed to support the broader application of acupuncture for NVP.

## Data Availability

The original contributions presented in the study are included in the article/**Supplementary Material**. Further inquiries can be directed to the corresponding author.
