# Supplementary material for: Acupuncture for nausea and vomiting during pregnancy: an overview of systematic reviews
Source: Front Endocrinol (Lausanne). 2026 Aug 7;17:1886667. doi: 10.3389/fendo.2026.1886667 (PMC13493242; doi:10.3389/fendo.2026.1886667)
Supplement: Supplementary file 1 [file DataSheet1.pdf]

Supplementary Material

1 Supplementary Data

Search Strategy

Search date: 2026.3.5

Pubmed: 20

Search	Query
#1	(((("Pregnancy"[Mesh]) OR (Pregnancies[Title/Abstract])) OR (Gestation[Title/Abstract])) AND ((((("Nausea"[Mesh]) OR (Antiemetics[Title/Abstract])) OR (Emetics[Title/Abstract])) OR (((("Vomiting"[Mesh])) OR (Antiemetics[Title/Abstract])) OR (Emetics[Title/Abstract]))) OR (("Morning Sickness"[Mesh]) OR (Sickness, Morning[Title/Abstract])))) OR ((("Hyperemesis Gravidarum"[Mesh]) OR (Pernicious Vomiting of Pregnancy[Title/Abstract])) OR (Pregnancy Pernicious Vomiting[Title/Abstract]))
#2	((((((((("Acupuncture"[Mesh])) OR (Pharmacopuncture[Title/Abstract])) OR ((((((((((("Acupuncture Therapy"[Mesh]) OR (Acupuncture Treatment[Title/Abstract])) OR (Acupuncture Treatments[Title/Abstract])) OR (Treatment, Acupuncture[Title/Abstract])) OR (Therapy, Acupuncture[Title/Abstract])) OR (Pharmacoacupuncture Treatment[Title/Abstract])) OR (Treatment, Pharmacoacupuncture[Title/Abstract])) OR (Pharmacoacupuncture Therapy[Title/Abstract])) OR (Therapy, Pharmacoacupuncture[Title/Abstract])) OR (Acupotomy[Title/Abstract])) OR (Acupotomies[Title/Abstract]))) OR (((((("Needles"[Mesh]) OR (Needle[Title/Abstract])) OR (Hypodermic Needles[Title/Abstract])) OR (Hypodermic Needle[Title/Abstract])) OR (Needle, Hypodermic[Title/Abstract])) OR (Needles, Hypodermic[Title/Abstract]))) OR (("Electroacupuncture"[Mesh]) OR (electro-acupuncture[Title/Abstract]))) OR (Acupoint application[Title/Abstract])) OR (Acupuncture point injection[Title/Abstract])) OR (("Acupuncture, Ear"[Mesh]) OR (((((((Acupunctures, Ear[Title/Abstract]) OR (Ear Acupunctures[Title/Abstract])) OR (Acupuncture, Auricular[Title/Abstract])) OR (Acupunctures, Auricular[Title/Abstract])) OR (Auricular Acupunctures[Title/Abstract])) OR (Auricular Acupuncture[Title/Abstract])) OR (Ear Acupuncture[Title/Abstract])))) OR (Moxibustion[Title/Abstract])
#3	#1 AND #2

Filters：	(meta-analysis OR systematic review)

Embase: 102

Search	Query
#1	'pregnancy'/exp
#2	'pregnancies':ab,ti
#3	'gestation':ab,ti
#4	#1 or #2 or #3
#5	'nausea'/exp
#6	'antiemetics':ab,ti
#7	'emetics':ab,ti
#8	#5 or #6 or #7
#9	'vomiting'/exp
#10	'antiemetics':ab,ti
#11	'emetics':ab,ti
#12	#9 or #10 or #11
#13	'morning sickness'/exp
#14	'sickness, morning':ab,ti
#15	#13 or #14
#16	#8 or #12 or #15

#17	#4 and #16
#18	'hyperemesis gravidarum'/exp
#19	'pernicious vomiting of pregnancy':ab,ti
#20	'pregnancy pernicious vomiting':ab,ti
#21	#18 or #19 or #20
#22	#17 or #21
#23	'acupuncture'/exp
#24	'pharmacopuncture':ab,ti
#25	#23 or #24
#26	'needle'/exp
#27	'needles':ab,ti
#28	'hypodermic needles':ab,ti
#29	'hypodermic needle':ab,ti
#30	'needle, hypodermic':ab,ti
#31	'needles, hypodermic':ab,ti
#32	#26 or #27 or #28 or #29 or #30 or #31
#33	'electroacupuncture'/exp
#34	'electro-acupuncture':ab,ti
#35	#33 or #34
#36	#25 or #32 or #35

#37	#22 and #36

Cochrane Central Register of Controlled Trials: 73

#1	MeSH descriptor: [Pregnancy] explode all trees
#2	(Pregnancies):ti,ab,kw OR (Gestation):ti,ab,kw
#3	#1 OR #2
#4	MeSH descriptor: [Nausea] explode all trees
#5	(Antiemetics):ti,ab,kw OR (Emetics):ti,ab,kw
#6	#4 OR #5
#7	MeSH descriptor: [Vomiting] explode all trees
#8	(Antiemetics):ti,ab,kw OR (Emetics):ti,ab,kw
#9	#7 OR #8
#10	MeSH descriptor: [Morning Sickness] explode all trees
#11	(Sickness, Morning):ti,ab,kw
#12	#10 OR #11
#13	#6 OR #9 OR #12
#14	#3 AND #13
#15	MeSH descriptor: [Hyperemesis Gravidarum] explode all trees
#16	(Pernicious Vomiting of Pregnancy):ti,ab,kw OR (Pregnancy Pernicious Vomiting):ti,ab,kw
#17	#15 OR #16

#18	#14 OR #17
#19	MeSH descriptor: [Acupuncture] explode all trees
#20	(Pharmacopuncture):ti,ab,kw
#21	#19 OR #20
#22	MeSH descriptor: [Acupuncture Therapy] explode all trees
#23	(Acupuncture Treatment):ti,ab,kw OR (Acupuncture Treatments):ti,ab,kw OR (Treatment, Acupuncture):ti,ab,kw OR (Therapy, Acupuncture):ti,ab,kw OR (Pharmacoacupuncture Treatment):ti,ab,kw OR (Treatment, Pharmacoacupuncture):ti,ab,kw OR (Pharmacoacupuncture Therapy):ti,ab,kw OR (Therapy, Pharmacoacupuncture):ti,ab,kw OR (Acupotomy):ti,ab,kw OR (Acupotomies):ti,ab,kw
#24	#22 OR #23
#25	MeSH descriptor: [Needles] explode all trees
#26	(Needle):ti,ab,kw OR (Needle, Hypodermic):ti,ab,kw OR (Hypodermic Needles):ti,ab,kw OR (Hypodermic Needle):ti,ab,kw OR (Needles, Hypodermic):ti,ab,kw
#27	#25 OR #26
#28	MeSH descriptor: [Electroacupuncture] explode all trees
#29	(Electro-acupuncture):ti,ab,kw
#30	#28 OR #29
#31	MeSH descriptor: [Acupuncture, Ear] explode all trees
#32	(Acupunctures, Auricular):ti,ab,kw OR (Acupuncture, Auricular):ti,ab,kw OR (Auricular Acupuncture):ti,ab,kw OR (Auricular Acupunctures):ti,ab,kw OR (Ear Acupunctures):ti,ab,kw OR (Auricular Acupunctures):ti,ab,kw OR (Acupunctures, Ear):ti,ab,kw
#33	#31 OR #32

#34	#21 OR #24 OR #27 OR #30 OR #33
#35	#18 AND #34

Web of Science: 40

#1	TS = (Pregnancy OR Pregnancies OR Gestation)
#2	TS = (Nausea OR Vomiting OR Antiemetics OR Emetics OR Morning Sickness OR Sickness, Morning)
#3	#1 AND #2
#4	TS = (Hyperemesis Gravidarum OR Pernicious Vomiting of Pregnancy OR Pregnancy Pernicious Vomiting)
#5	#3 OR #4
#6	TS = (Acupuncture OR Pharmacopuncture OR Acupuncture Therapy OR Acupuncture Treatment OR Acupuncture Treatments OR Treatment, Acupuncture OR Therapy, Acupuncture OR Pharmacoacupuncture Treatment OR Treatment, Pharmacoacupuncture OR Pharmacoacupuncture Therapy OR Therapy, Pharmacoacupuncture OR Acupotomy OR Acupotomies OR Needles OR Hypodermic Needles OR Hypodermic Needle OR Needle, Hypodermic OR Needles, Hypodermic OR Electroacupuncture OR Electro-acupuncture OR Moxibustion OR Acupoint application OR Acupuncture point injection OR Acupuncture, Ear OR Acupunctures, Ear OR Ear Acupunctures OR Acupuncture, Auricular OR Acupunctures, Auricular OR Auricular Acupunctures OR Auricular Acupuncture OR Ear Acupuncture)
#7	#5 AND #6
#8	TS = (Meta Analysis OR Systematic Review)
#9	#7 AND #8

CINAHL: 11

#1	('pregnancy') OR XB ('pregnancies' OR 'gestation')
#2	('nausea') OR XB ('antiemetics' OR 'emetics')
#3	('vomiting') OR XB ('antiemetics' OR 'emetics')
#4	('morning sickness') OR XB ('sickness, morning')
#5	('hyperemesis gravidarum') OR XB ('pernicious vomiting of pregnancy' OR 'pregnancy pernicious vomiting')
#6	(#2 OR #3 OR #4 OR #5)
#7	#1 AND #6
#8	('acupuncture therapy') OR XB ('acupuncture treatment' OR 'acupuncture treatments' OR 'treatment,acupuncture' OR 'therapy,acupuncture' OR 'pharmacoacupuncture treatment' OR 'treatment, pharmacoacupuncture' OR 'pharmacoacupuncture therapy' OR 'therapy, pharmacoacupuncture' OR 'acupotomy' OR 'acupotomies')
#9	('acupuncture analgesia') OR XB ('analgesia, acupuncture' OR 'acupuncture anesthesia' OR 'anesthesia, acupuncture')
#10	('acupuncture, ear') OR XB ('acupunctures, ear' OR 'ear acupunctures' OR 'acupuncture, auricular' OR 'acupunctures, auricular' OR 'auricular acupunctures' OR 'auricular acupuncture' OR 'ear acupuncture')
#11	('acupuncture points') OR XB ('acupuncture point' OR 'point, acupuncture' OR 'points, acupuncture' OR 'acupoints' OR 'acupoint')
#12	('needles') OR XB ('needle' OR 'hypodermic needles' OR 'hypodermic needle' OR 'needles,hypodermic' OR 'needle,hypodermic')
#13	('moxibustion')
#14	('electroacupuncture')
#15	#8 OR #9 OR #10 OR #11 OR #12 OR #13 OR #14
#16	#7 AND #15

#17	('Meta Analysis' OR 'Systematic Review')
#18	#16 AND #17

China National Knowledge Infrastructure: 5

#1	SU='妊娠'
#2	SU=('恶心'+'呕吐'+'恶阻'+'剧吐')
#3	#1 AND #2
#4	SU=('针刺'+'干针'+'电针'+'耳针'+'耳穴'+'火针'+'手针'+'体针'+'毫针'+'温针'+'头针'+'腹针'+'腕踝针'+'眼针'+'水针'+'芒针'+'埋针'+'梅花针'+'艾灸'+'穴位注射'+'穴位贴敷')
#5	#3 AND #4
#6	SU=('Meta 分析'+'荟萃分析'+'系统评价'+'系统综述')
#7	#5 AND #6

Wanfang Database: 27

#1	主题:(妊娠)
#2	主题:(恶心 OR 呕吐 OR 恶阻 OR 剧吐)
#3	#1 AND #2
#4	主题:(针刺 OR 干针 OR 电针 OR 耳针 OR 耳穴 OR 火针 OR 手针 OR 体针 OR 毫针 OR 温针 OR 头针 OR 腹针 OR 腕踝针 OR 眼针 OR 水针 OR 芒针 OR 埋针 OR 梅花针 OR 艾灸 OR 穴位注射 OR 穴位贴敷)
#5	#3 AND #4

#6	主题:(Meta 分析 or 荟萃分析 or 系统评价 or 系统综述)
#7	#5 AND #6

Chinese Science and Technology Journals Database: 4

#1	M=妊娠
#2	(M=恶心+M=呕吐+M=恶阻+M=剧吐)
#3	#1 AND #2
#4	(M=针刺+M=干针+M=电针+M=耳针+M=耳穴+M=火针+M=手针+M=体针+M=毫针+M=温针+M=头针+M=腹针+M=腕踝针+M=眼针+M=水针+M=芒针+M=埋针+M= 梅花针+M= 艾灸+M= 穴位注射+M= 穴位贴敷)
#5	#3 AND #4
#6	(U= Meta 分析+U=荟萃分析+U=系统评价+U=系统综述)
#7	#5 AND #6

Chinese Biomedical Literature Database: 6

#1	"恶心"[摘要:智能] OR "呕吐"[摘要:智能] OR "恶阻"[摘要:智能] OR "剧吐"[摘要:智能]
#2	"妊娠"[摘要:智能]
#3	#1 AND #2
#4	"针刺"[摘要:智能] OR "干针"[摘要:智能] OR "电针"[摘要:智能] OR "耳针"[摘要:智能] OR "耳穴"[摘要:智能] OR "火针"[摘要:智能] OR "手针"[摘要:智能] OR "体针"[摘要:智能] OR "毫针"[摘要:智能] OR "温针"[摘要:智能] OR "头针"[摘要:智能] OR "腹针"[摘要:智能] OR "腕踝针"[摘要:智能] OR "眼针"[摘要:智能] OR "水针"[摘要:智能] OR "芒针"[摘要:智能] OR "埋针"[摘

	要:智能] OR "梅花针"[摘要:智能] OR "艾灸"[摘要:智能] OR "穴位贴敷"[摘要:智能] OR "穴位注射"[摘要:智能]
#5	#3 AND #4
#6	"Meta 分析"[摘要:智能] OR "荟萃分析"[摘要:智能] OR "系统评价"[摘要:智能] OR "系统综述"[摘要:智能]
#7	#5 AND #6

2 Supplementary Figures and Tables

2.1 Supplementary Figure 1. Flow diagram of the study selection process.

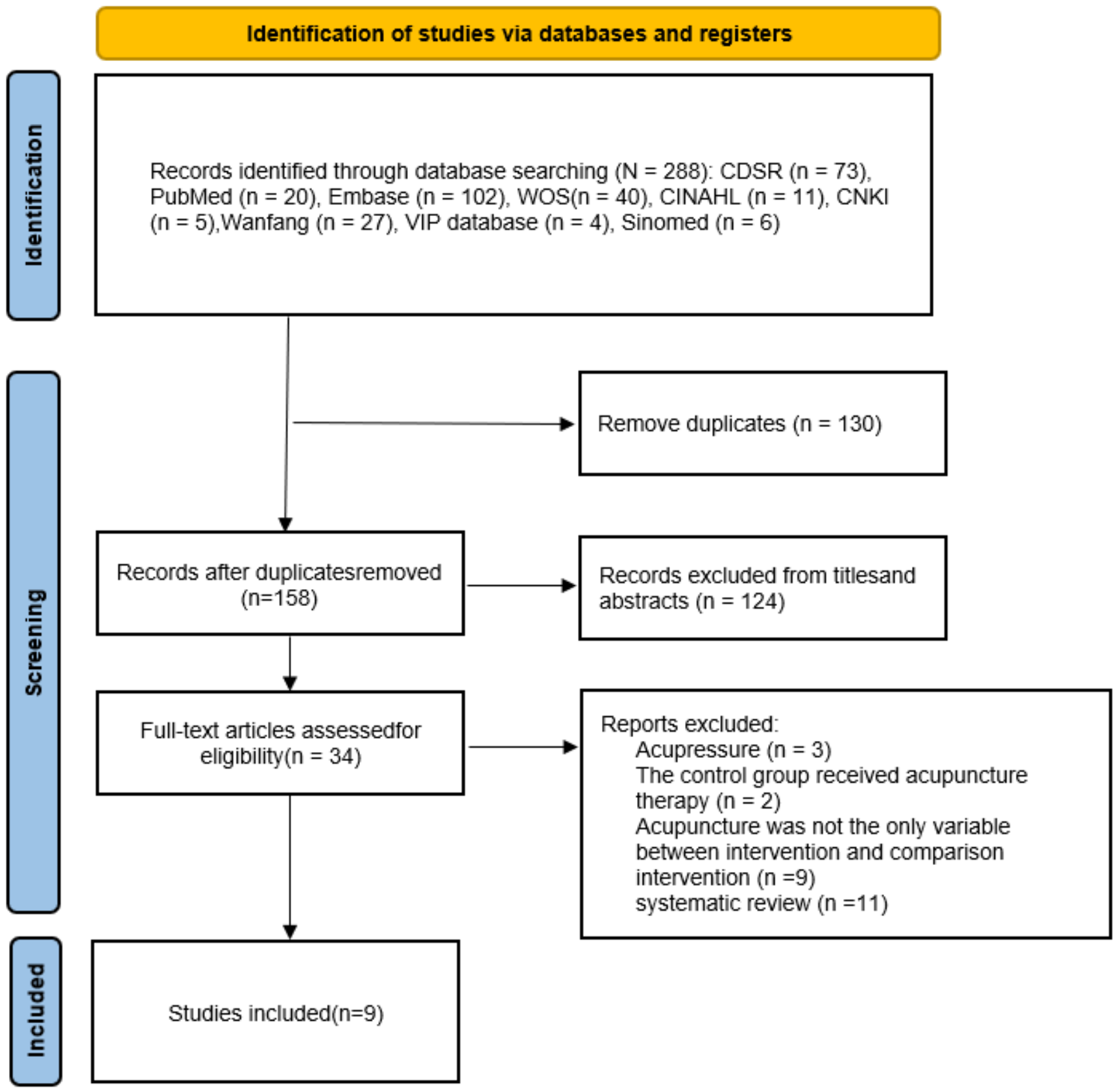

2.2 Supplementary Figure 2. Evidence map of the methodological quality.

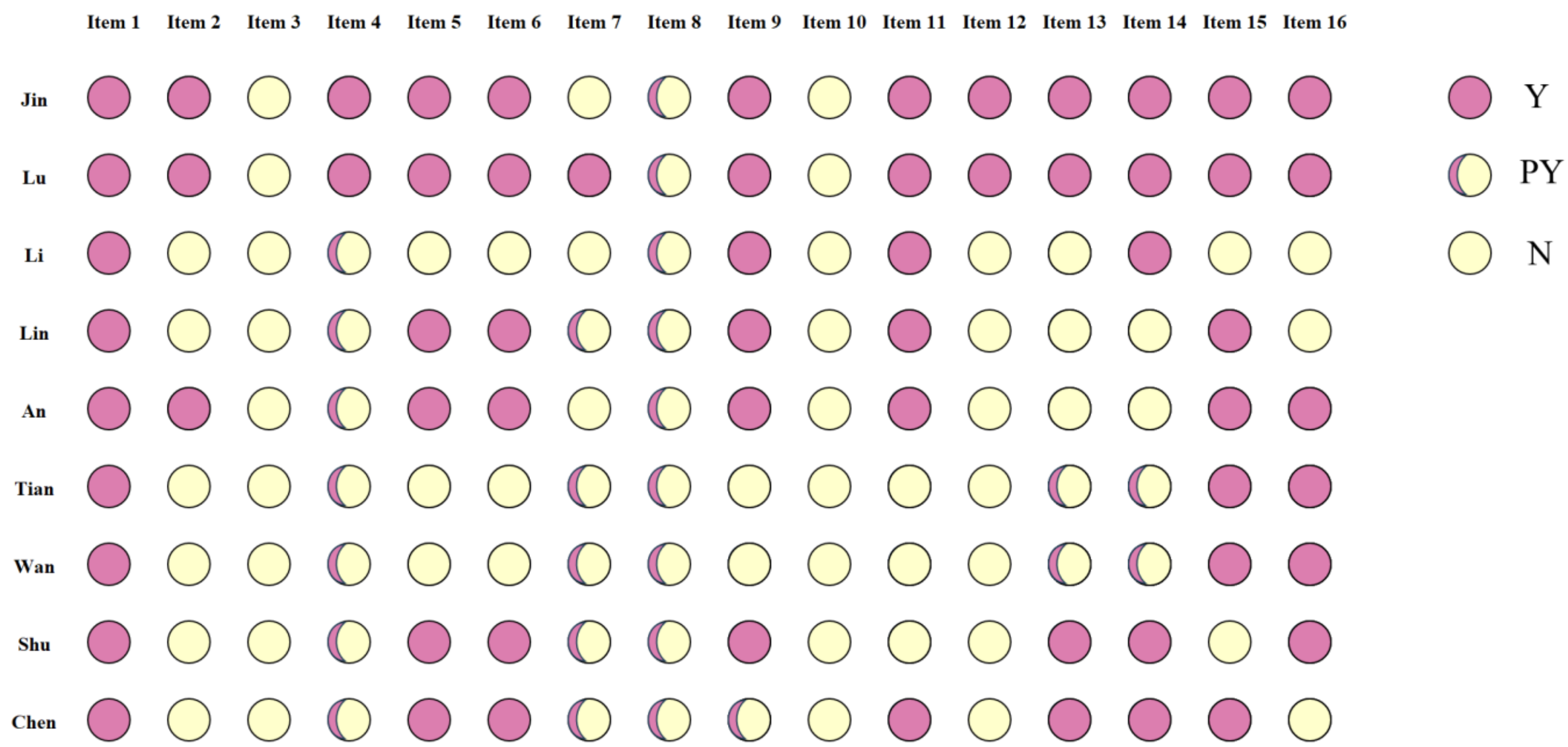

2.3 Supplementary Figure 3. Reporting quality assessment by PRISMA-A.

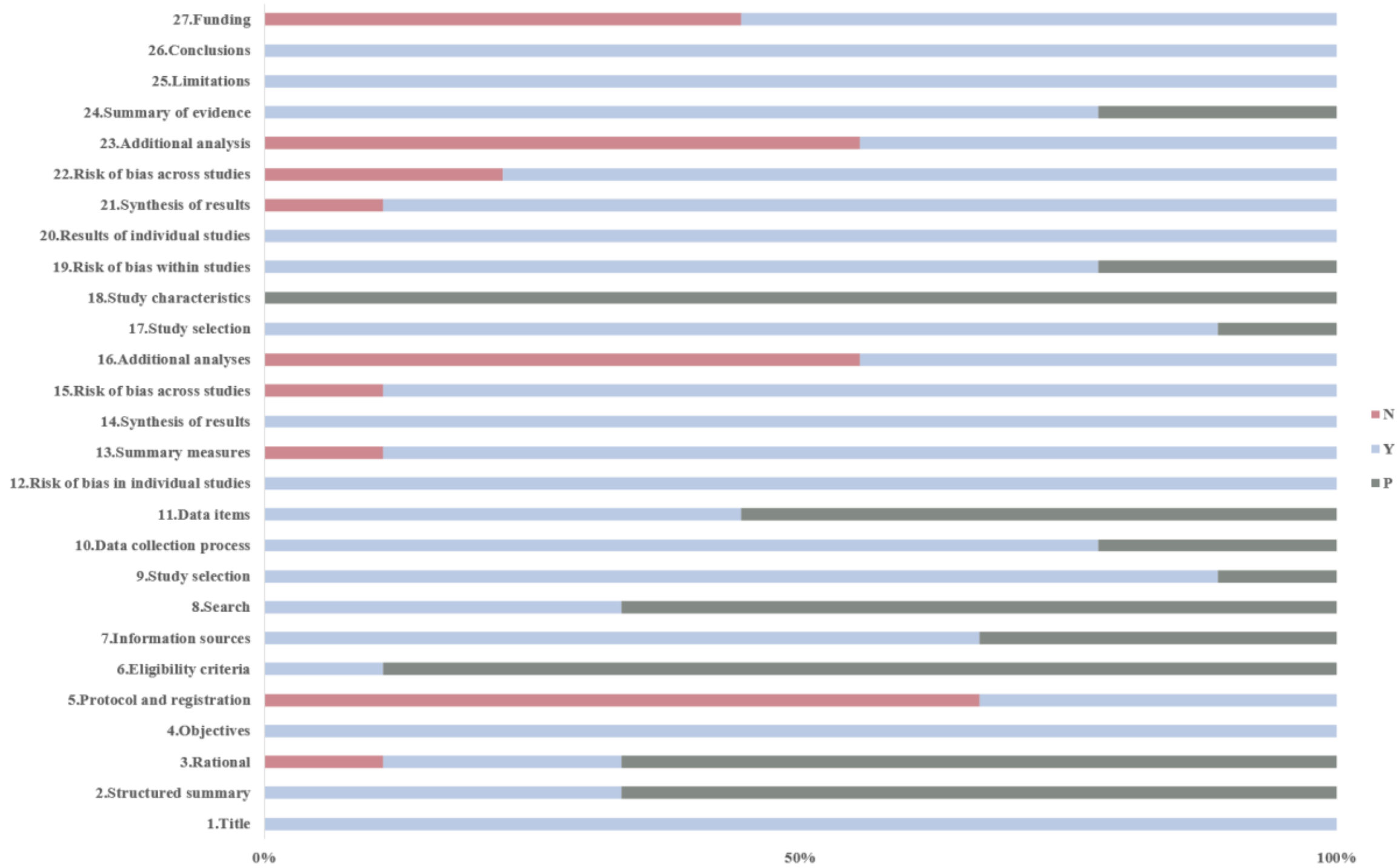

2.4 Supplementary Figure 4. Overall Reporting Quality Assessment of Each Study Included in the Systematic Review

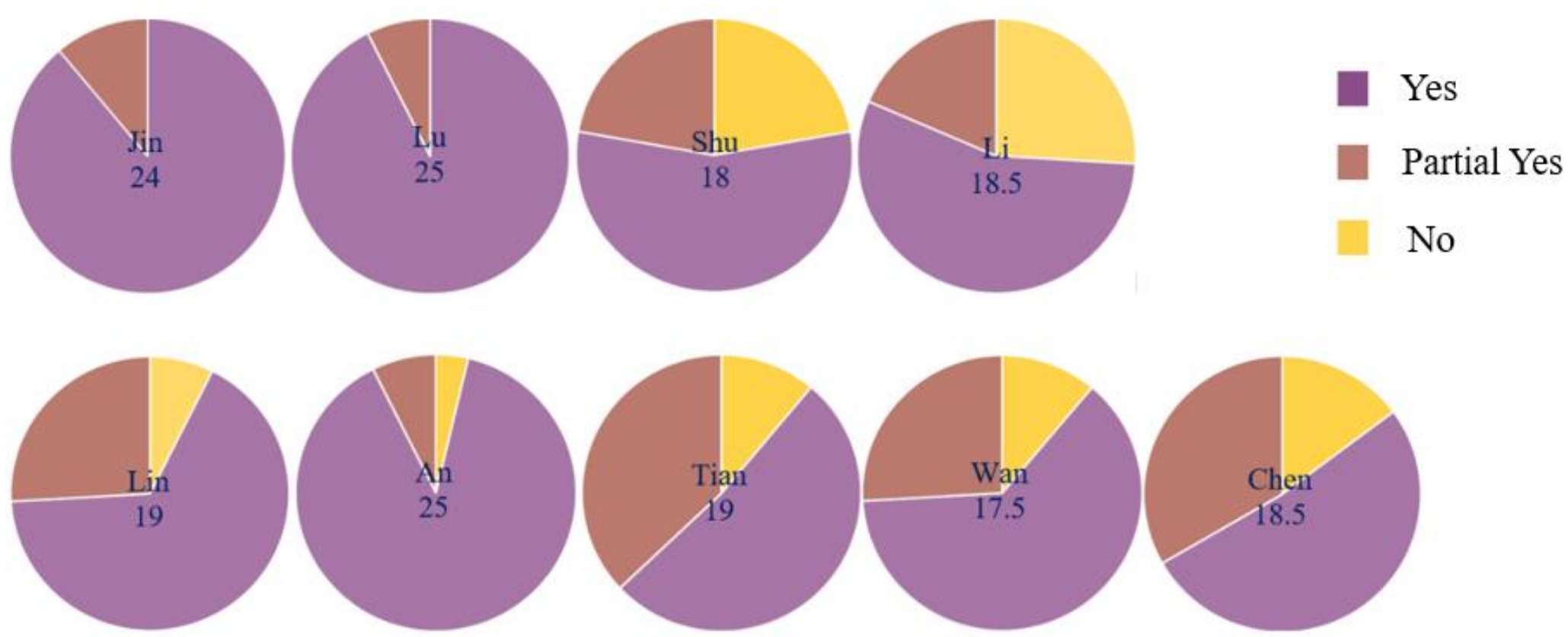

2.5 Supplementary Figure 5. Matrix diagram of GROOVE

A

Number of columns (number of reviews)	c	9
Number of rows (number of index publications)	r	110
Number of included primary studies (including double counting)	N	142
Covered area	N/(rc)	14.34%
Corrected covered area	(N-r)/(rc-r)	3.64%
Interpretation of overlap	**Slight overlap**	
Structural Zeros	X	2
Corrected covered area<br>(adjusting by structural zeros)	(N-r)/(rc-r-X)	3.64%

B

N° of non-overlapped primary studies	In 1 SR	85
Number of overlapped primary studies	In 2 SRs	16
	In 3 SRs	7
	In 4 SRs	1

C

	Lu2021	Jin2024	Wan2020	Chen2022	Lin2019	Li2017	Tian2022	Shu2022
Jin2024	32.3%							
Wan2020	0.0%	0.0%						
Chen2022	0.0%	0.0%	8.0%					
Lin2019	0.0%	0.0%	0.0%	0.0%				
Li2017	28.6%	24.1%	0.0%	0.0%	0.0%			
Tian2022	0.0%	0.0%	0.0%	0.0%	0.0%	0.0%		
Shu2022	0.0%	0.0%	0.0%	0.0%	0.0%	0.0%	12.5%	
An2025	4.1%	3.4%	2.2%	16.3%	2.3%	2.2%	2.6%	2.1%

**36**	=	**Total nodes (pairs of reviews)**
30	=	Slight overlap (<5%)
1	=	Moderate overlap (5% to <10%)
1	=	High overlap (10% to <15%)
4	=	Very High overlap (≥15%)

(A) Overall overlap results using the GROOVE tool; (B) Number of non-overlapped and overlapped primary studies; (C) Graphical representation of overlap for overviews.

2.6 Supplementary Table 1. Basic characteristics of the included systematic reviews and meta-analyses.

Study	Country	Year	N/n	Intervention	Control	Assessment Tool	Outcome	Safety	Funding
**Li**	China	2017	N = 11/n = 1043	Acupuncture	Usual care	Cochrane risk of bias tool	②③⑧⑭⑮	Unknown	Yes
**Lin**	China	2019	N = 10/n = 834	AIT + usual care	Usual care	Cochrane risk of bias tool; Modified Jadad score scale	②⑧⑲	Unknown	Yes
**Wan**	China	2020	N = 12/n = 1053	AAT + usual care	Usual care	Cochrane risk of bias tool; Modified Jadad score scale	⑧⑯⑰⑲	Unknown	Unknown
**Lu**	China	2021	N = 16/n = 1043	Acupuncture	Usual care; SA；	Cochrane risk of bias tool	②③⑧⑪⑫⑬⑭⑮	No	No
**Tian**	China	2022	N = 5/n = 359	APA + usual care	Usual care	Modified Jadad score scale	⑧⑲	Unknown	Unknown
**Shu**	China	2022	N = 13/n = 1026	APA	Usual care; SA;	Cochrane risk of bias tool	①⑧	Unknown	No
**Chen**	China	2022	N = 16/n = 1150	AAT; AAT + TAU	Usual care	Cochrane risk of bias tool; Modified Jadad score scale	②⑧⑪⑫⑳	No	Unknown
**Jin**	China	2024	N = 24/n = 2390	Acupuncture; Acupuncture + usual care	SA; usual care	Cochrane risk of bias tool	①②③④⑤⑥⑦⑧⑨⑩	Yes	Yes
**An**	China	2025	N = 35/n = 2672	Acupuncture + usual care	Usual care	Cochrane risk of bias tool	①⑧	Unknown	Yes

Note: Acupuncture: Acupuncture; Acupoint application therapy: AAT; Auricular acupressure: APA; acupoint injection therapy: AIT; Sham acupuncture: SA;

2.7 Supplementary Table 2. Methodological quality assessment of the included systematic reviews

	Li	Lin	Wan	Lu	Tian	Shu	Chen	Jin	An
**Item 1**	Y	Y	Y	Y	Y	Y	Y	Y	Y
**Item 2**	N	N	N	Y	N	N	N	Y	Y
**Item 3**	N	N	N	N	N	N	N	N	N
**Item 4**	PY	PY	PY	Y	PY	PY	PY	Y	PY
**Item 5**	N	Y	N	Y	N	Y	Y	Y	Y
**Item 6**	N	Y	N	Y	N	Y	Y	Y	Y
**Item 7**	N	PY	PY	Y	PY	PY	PY	N	N
**Item 8**	PY	PY	PY	PY	PY	PY	PY	PY	PY
**Item 9**	Y	Y	N	Y	N	Y	PY	Y	Y
**Item 10**	N	N	N	N	N	N	N	N	N
**Item 11**	Y	Y	N	Y	N	N	Y	Y	Y
**Item 12**	N	N	N	Y	N	N	N	Y	N
**Item 13**	N	N	PY	Y	PY	Y	Y	Y	N
**Item 14**	Y	N	PY	Y	PY	Y	Y	Y	N
**Item 15**	N	Y	Y	Y	Y	N	Y	Y	Y
**Item 16**	N	N	Y	Y	Y	Y	N	Y	Y
**Ranking of quality**	**Critically low**	**Critically low**	**Critically low**	**Moderate**	**Critically low**	**Critically low**	**Low**	**Low**	**Critically low**

AMSTAR: A Measurement Tool to Assess Systematic Reviews. N: no; PY: partial yes; Y: yes.

2.8 Supplementary Table 3. Methodological quality assessment of the included systematic reviews

Study	Phase1	Phase2				Phase3
		Study eligibility criteria	Identification and selection of studies	Data collection and study appraisal	Synthesis and findings	Risk of bias in the review
**Li**	L	H	H	U	H	H
**Lin**	L	H	H	L	H	H
**Wan**	L	H	H	L	H	H
**Lu**	L	L	L	L	L	L
**Tian**	L	H	H	H	H	H
**Shu**	L	L	H	L	U	H
**Chen**	L	H	H	H	L	H
**Jin**	L	L	L	L	L	L
**An**	L	L	L	L	L	L

Note: L, low risk; H, high risk; U, unclear risk.

2.9 Supplementary Table 4. Reporting quality assessment by PRISMA-A.

Item	Li	Lin	Wan	Lu	Tian	Shu	Chen	Jin	An
**Item 1**	Y	Y	Y	Y	Y	Y	Y	Y	Y
**Item 2**	P	P	P	Y	P	P	P	Y	Y
**Item 3**	N	P	P	Y	P	P	P	P	Y
**Item 4**	Y	Y	Y	Y	Y	Y	Y	Y	Y
**Item 5**	N	N	N	Y	N	N	N	Y	Y
**Item 6**	P	P	P	P	P	P	P	P	P
**Item 7**	Y	P	P	Y	Y	Y	Y	Y	Y
**Item 8**	P	P	P	Y	P	P	P	Y	Y
**Item 9**	Y	Y	Y	Y	P	Y	Y	Y	Y
**Item 10**	Y	Y	Y	Y	P	Y	P	Y	Y
**Item 11**	Y	P	P	Y	P	N	P	Y	Y
**Item 12**	Y	Y	Y	Y	Y	Y	Y	Y	Y
**Item 13**	Y	Y	Y	Y	Y	Y	Y	Y	Y
**Item 14**	Y	Y	Y	Y	Y	Y	Y	Y	Y
**Item 15**	N	Y	Y	Y	Y	Y	Y	Y	Y
**Item 16**	N	Y	N	Y	N	N	N	Y	Y
**Item 17**	P	Y	Y	Y	Y	Y	Y	Y	Y
**Item 18**	P	P	P	P	P	P	P	P	P
**Item 19**	Y	Y	Y	Y	P	Y	P	Y	Y
**Item 20**	Y	Y	Y	Y	Y	Y	Y	Y	Y
**Item 21**	Y	Y	Y	Y	Y	N	Y	Y	Y
**Item 22**	N	Y	Y	Y	Y	N	Y	Y	Y
**Item 23**	N	Y	N	Y	N	N	N	Y	Y
**Item 24**	Y	Y	P	Y	P	Y	Y	Y	Y
**Item 25**	Y	Y	Y	Y	Y	Y	Y	Y	Y
**Item 26**	Y	Y	Y	Y	Y	Y	Y	Y	Y
**Item 27**	N	N	Y	Y	Y	Y	N	Y	N

N: no; P: partial yes; Y: yes.

2.10 Supplementary Table 5. Meta-analyses and GRADE evidence evaluation(Intervention: Acupuncture)

Outcome	Study	Intervention	Control	Included RCTs (participants)	Effect (95% CI)	Quality assessment					Quality of evidence
						Risk of bias	Inconsistency	Indirectness	Imprecision	Publication bias	
**Overall response rate**	An	Acupuncture + Usual care	Usual care	7(518)	RR=1.20 (1.01, 1.42)	-1[a]	-1[b]	0	0	-1[d]	Very Low
	Li	Acupuncture + ivgtt	Medications ＋ ivgtt	2(104)	OR=7.03 (4.47, 11.06)	-1[a]	0	0	0	-1[d]	Low
	Li	Acupuncture	ivgtt	3(219)	OR=7.03 (4.47, 11.06)	-1[a]	0	0	0	-1[d]	Low
	Lu	Acupuncture	Ivgtt; Medications	2(113)	OR=8.11 (5.29, 12.43)	-1[a]	0	0	0	-1[d]	Low
	Lu	Acupuncture + ivgtt; Acupuncture + medications	Ivgtt; Medications	8(500)	OR=8.11 (5.29, 12.43)	-1[a]	0	0	0	-1[d]	Low
**Ineffective rates**	Jin	Acupuncture	Medications	5(357)	RR=0.50 (0.30, 0.81)	-1[a]	0	0	0	-1[d]	Low
**PUQE**	Jin	Acupuncture + Medications	Medications	6(523)	MD=-1.95 (-3.08, -0.81)	-1[a]	-1[b]	0	0	-1[d]	Very Low
	Jin	Acupuncture	Medications	3(279)	MD=-0.80 (-3.06, 1.46)	-1[a]	-1[b]	0	-1[d]	-1[d]	Very Low
	An	Acupuncture + Usual care	Usual care	3(220)	MD=-2.67 (-4.25, -1.08)	-1[a]	-1[b]	0	0	-1[d]	Very Low
**Anxiety**	Jin	Acupuncture + Medications	Medications	2(246)	MD=-1.46 (-3.80, 0.87)	-1[a]	-1[b]	0	-1[d]	-1[d]	Very Low
	Jin	Acupuncture	SA	2(180)	MD=0.78 (-1.13, 2.69)	-1[a]	0	0	-1[d]	-1[d]	Very Low
**Depression**	Jin	Acupuncture	SA	2(246)	MD=-1.24 (-2.61, 0.13)	-1[a]	0	0	-1[d]	-1[d]	Very Low
	Jin	Acupuncture	SA	2(180)	MD=0.20 (-2.05, 2.46)	-1[a]	0	0	-1[d]	-1[d]	Very Low
**Ketonuria negative rates**	Jin	Acupuncture + Medications	Medications	6(431)	RR=1.32 (1.14, 1.53)	-1[a]	0	0	0	-1[d]	Low
	Jin	Acupuncture	Medications	4(330)	RR=1.27 (0.85, 1.90)	-1[a]	-1[b]	0	-1[d]	-1[d]	Very Low
	Lu	Acupuncture + Medications	Medications	3(203)	RR=1.36 (1.15, 1.60)	-1[a]	0	0	-1[d]	-1[d]	Very Low
	Li	Acupuncture + ivgtt	Ivgtt	1(40)	OR=4.85 (0.86, 27.22)	-1[a]	-1[b]	0	-1[d]	-1[d]	Very Low
**NVPQOL**	Jin	Acupuncture + Medications	Medications	3(301)	MD=-10.89 (-13.92, -7.86)	-1[a]	0	0	0	-1[d]	Low
**length of stay (LOS)**	Jin	Acupuncture + Medications	Medications	4(267)	MD=-2.89 (-4.01, -1.77)	-1[a]	0	0	0	-1[d]	Low
	Lu	Acupuncture + Medications	Medications	2(104)	MD=-3.78 (-5.39, -2.16)	-1[a]	-1[b]	0	0	-1[d]	Very Low
	Li	Acupuncture + ivgtt	Ivgtt	2(104)	SMD=-2.32 (-3.01, -1.63)	-1[a]	-1[b]	0	0	-1[d]	Very Low
**Chinese Medicine Syndrome Scale (CMS) score**	Jin	Acupuncture + Medications	Medications	4(292)	SMD=-0.67 (-0.99, -0.36)	-1[a]	0	0	0	-1[d]	Low
**Adverse events**	Jin	Acupuncture	SA	2(434)	RR=0.64 (0.28, 1.46)	-1[a]	0	0	-1[d]	-1[d]	Very Low
	Jin	Acupuncture	Medications	2(236)	RR=0.67 (0.17, 2.69)	-1[a]	0	0	-1[d]	-1[d]	Very Low
**Improvement rate of nausea and vomiting symptoms**	Lu	Acupuncture + Medications	Medications	2(153)	OR=26.44 (3.54, 197.31)	-1[a]	0	0	-1[d]	-1[d]	Very Low
**Improvement in fatigue**	Lu	Acupuncture + Medications	Medications	2(122)	RR=1.01 (0.89, 1.14)	-1[a]	0	0	-1[d]	-1[d]	Very Low
**Pregnancy termination rate**	Li	Acupuncture + ivgtt	Ivgtt	1(40)	OR=0.14 (0.02, 0.75)	-1[a]	-1[b]	0	-1[d]	-1[d]	Very Low
	Lu	Acupuncture + Medications	Medications	1(40)	RR=0.26 (0.09, 0.74)	-1[a]	0	0	-1[d]	-1[d]	Very Low
	Lu	Acupuncture	Medications	1(95)	RR=0.26 (0.09, 0.74)	-1[a]	0	0	-1[d]	-1[d]	Very Low
**Serum potassium**	Li	Acupuncture + ivgtt	Ivgtt	1(40)	MD=0.11 (-0.04, 0.25)	-1[a]	0	0	-1[d]	-1[d]	Very Low
	Li	Acupuncture	Ivgtt	1(95)	MD=0.11 (-0.04, 0.25)	-1[a]	0	0	-1[d]	-1[d]	Very Low
	Lu	Acupuncture	Medications	1(95)	MD=0.11 (-0.04, 0.25)	-1[a]	0	0	-1[d]	-1[d]	Very Low
	Lu	Acupuncture + Medications	Medications	1(40)	MD=0.11 (-0.04, 0.25)	-1[a]	0	0	-1[d]	-1[d]	Very Low
**Improvement in dietary intake/food consumption**	Lu	Acupuncture	Medications	1(81)	MD=0.11 (-0.04, 0.25)	-1[a]	0	0	-1[d]	-1[d]	Very Low
	Lu	Acupuncture + Medications	Medications	2(166)	MD=0.11 (-0.04, 0.25)	-1[a]	0	0	-1[d]	-1[d]	Very Low

Note: RR, relative risk; SMD, standardized mean difference; OR, odds ratio; MD, mean difference. a: The original studies had methodological problems related to blinding, randomization, allocation concealment, or other aspects of study design. b: Substantial heterogeneity was observed among the included studies. c: The included studies had small sample sizes and wide confidence intervals. d: Publication bias was possible, substantial publication bias was mentioned in the article, the funnel plot was asymmetric, or publication bias was not assessed.

Acupuncture: Acupuncture; Intravenous fluid therapy: ivgtt; Sham acupuncture: SA;

2.11 Supplementary Table 6 Meta-analyses and GRADE evidence evaluation(Intervention: Acupuncture and Moxibustion)

Outcome	Study	Intervention	Control	Included RCTs (participants)	Effect (95% CI)	Quality assessment					Quality of evidence
						Risk of bias	Inconsistency	Indirectness	Imprecision	Publication bias	
**Overall response rate**	Lu	AMT	Medications	1(100)	OR=8.11 (5.29, 12.43)	-1[a]	0	0	0	-1[d]	Low
	Lu	AMT＋ ivgtt	Ivgtt	1(70)	OR=8.11 (5.29, 12.43)	-1[a]	0	0	0	-1[d]	Low
	Li	AMT	Ivgtt	1(70)	OR=7.03 (4.47, 11.06)	-1[a]	0	0	0	-1[d]	Low

OR, odds ratio;
Intravenous fluid therapy: ivgtt; Acupuncture and moxibustion therapy: AMT;

2.12 Supplementary Table 7 Meta-analyses and GRADE evidence evaluation(Intervention: Acupuncture modalities)

Outcome	Study	Intervention	Control	Included RCTs (participants)	Effect (95% CI)	Quality assessment					Quality of evidence
						Risk of bias	Inconsistency	Indirectness	Imprecision	Publication bias	
**Overall response rate**	An	MWM + Usual care	Usual care	2(175)	RR=1.41 (1.14, 1.73)	-1[a]	0	0	0	-1[d]	Low
	An	AIT + Usual care	Usual care	3(269)	RR=1.20 (1.09, 1.32)	-1[a]	0	0	0	-1[d]	Low
	An	AAT + Usual care	Usual care	13(919)	RR=1.19 (1.13, 1.25)	-1[a]	0	0	0	-1[d]	Low
	Tian	APA + Usual care	Usual care	5(359)	OR=5.01 (2.50, 10.07)	-1[a]	0	0	0	-1[d]	Low
	Lin	AIT + ivgtt	Ivgtt	7(524)	OR=4.95 (2.66, 9.23)	-1[a]	0	0	0	-1[d]	Low
	Lin	AIT	Ivgtt	3(310)	OR=4.95 (2.66, 9.23)	-1[a]	0	0	0	-1[d]	Low
	Chen	AAT + Ivgtt	Ivgtt	8(596)	OR=4.66 (3.08, 7.03)	-1[a]	0	0	0	0	Moderate
	Chen	AAT	Ivgtt	4(240)	OR=4.66 (3.08, 7.03)	-1[a]	0	0	0	0	Moderate
	Wan	AAT + ivgtt	Ivgtt	6(424)	OR=7.49(4.64, 12.09)	-1[a]	0	0	0	-1[d]	Low
	Wan	AAT + TCM + ivgtt	Ivgtt + TCM	3(284)	OR=7.49(4.64, 12.09)	-1[a]	0	0	0	-1[d]	Low
**Cure rate**	Tian	AAT + Usual care	Usual care	5(359)	OR=2.42 (2.14, 5.45)	-1[a]	0	0	0	-1[d]	Low
	Lin	AIT + ivgtt	Ivgtt	7(524)	OR=4.18 (3.02, 5.78)	-1[a]	0	0	0	0	Moderate
	Lin	AIT	Ivgtt	3(310)	OR=4.18 (3.02, 5.78)	-1[a]	0	0	0	0	Moderate
	Wan	AAT + ivgtt	Ivgtt	2(272)	OR=3.25 (2.93, 4.52)	-1[a]	0	0	0	-1[d]	Low
	Wan	AAT + TCM + ivgtt	Usual care + TCM	1(102)	OR=3.25 (2.93, 4.52)	-1[a]	0	0	0	-1[d]	Low
**PUQE**	An	AIT + Usual care	Usual care	4(352)	MD=-2.30 (-2.82, -1.79)	-1[a]	-1[b]	0	0	-1[d]	Very Low
	An	AAT + Usual care	Usual care	3(224)	MD=-1.06 (-1.77, -0.35)	-1[a]	-1[b]	0	0	-1[d]	Very Low
**Recurrence rate**	Chen	AAT + ivgtt	Ivgtt	4(375)	OR=0.14 (0.08, 0.24)	-1[a]	0	0	0	-1[d]	Low
**Ketonuria negative rates**	Lin	AIT + ivgtt	Ivgtt	6(428)	OR=7.51 (4.05, 13.90)	-1[a]	0	0	0	-1[d]	Low
	Lin	AIT	Ivgtt	2(210)	OR=7.51 (4.05, 13.90)	-1[a]	0	0	0	-1[d]	Low
	Chen	AAT + ivgtt	Ivgtt	2(186)	OR=3.78 (1.86, 7.67)	-1[a]	0	0	0	-1[d]	Low
	Chen	AAT	Ivgtt	1(60)	OR=3.78 (1.86, 7.67)	-1[a]	0	0	0	-1[d]	Low
**Improvement rate of nausea and vomiting symptoms**	Shu	APA	SA	1(128)	P<0.001	-1[a]	-1[b]	0	-1[d]	-1[d]	Very Low
	Shu	APA＋ivgtt	Ivgtt	9(646)	P<0.001	-1[a]	-1[b]	0	-1[d]	-1[d]	Very Low
	Shu	APA	Ivgtt	2(154)	P<0.001	-1[a]	-1[b]	0	-1[d]	-1[d]	Very Low

Note: RR, relative risk; SMD, standardized mean difference; OR, odds ratio; MD, mean difference. a: The original studies had methodological problems related to blinding, randomization, allocation concealment, or other aspects of study design. b: Substantial heterogeneity was observed among the included studies. c: The included studies had small sample sizes and wide confidence intervals. d: Publication bias was possible, substantial publication bias was mentioned in the article, the funnel plot was asymmetric, or publication bias was not assessed.

Intravenous fluid therapy: ivgtt; Acupoint application therapy: AAT; Auricular acupressure: APA; acupoint injection therapy: AIT; Sham acupuncture: SA; Traditional Chinese medicine: TCM; Warm needle moxibustion: MWM;
