# Supplementary material for: Acupuncture for nausea and vomiting during pregnancy: an overview of systematic reviews
Source: Front Endocrinol (Lausanne). 2026 Aug 7;17:1886667. doi: 10.3389/fendo.2026.1886667 (PMC13493242; doi:10.3389/fendo.2026.1886667)
Supplement: Supplementary file 2 [file SupplementaryFile1.docx]

Supplementary Material

# 1 Supplementary Data

# Search Strategy

Search date: 2026.3.5

**Pubmed: 20**

| Search | Query |
| --- | --- |
| #1 | **(((("Pregnancy"[Mesh]) OR (Pregnancies[Title/Abstract])) OR (Gestation[Title/Abstract])) AND ((((("Nausea"[Mesh]) OR (Antiemetics[Title/Abstract])) OR (Emetics[Title/Abstract])) OR (((("Vomiting"[Mesh])) OR (Antiemetics[Title/Abstract])) OR (Emetics[Title/Abstract]))) OR (("Morning Sickness"[Mesh]) OR (Sickness, Morning[Title/Abstract])))) OR ((("Hyperemesis Gravidarum"[Mesh]) OR (Pernicious Vomiting of Pregnancy[Title/Abstract])) OR (Pregnancy Pernicious Vomiting[Title/Abstract]))** |
| #2 | **((((((((("Acupuncture"[Mesh])) OR (Pharmacopuncture[Title/Abstract])) OR ((((((((((("Acupuncture Therapy"[Mesh]) OR (Acupuncture Treatment[Title/Abstract])) OR (Acupuncture Treatments[Title/Abstract])) OR (Treatment, Acupuncture[Title/Abstract])) OR (Therapy, Acupuncture[Title/Abstract])) OR (Pharmacoacupuncture Treatment[Title/Abstract])) OR (Treatment, Pharmacoacupuncture[Title/Abstract])) OR (Pharmacoacupuncture Therapy[Title/Abstract])) OR (Therapy, Pharmacoacupuncture[Title/Abstract])) OR (Acupotomy[Title/Abstract])) OR (Acupotomies[Title/Abstract]))) OR (((((("Needles"[Mesh]) OR (Needle[Title/Abstract])) OR (Hypodermic Needles[Title/Abstract])) OR (Hypodermic Needle[Title/Abstract])) OR (Needle, Hypodermic[Title/Abstract])) OR (Needles, Hypodermic[Title/Abstract]))) OR (("Electroacupuncture"[Mesh]) OR (electro-acupuncture[Title/Abstract]))) OR (Acupoint application[Title/Abstract])) OR (Acupuncture point injection[Title/Abstract])) OR (("Acupuncture, Ear"[Mesh]) OR (((((((Acupunctures, Ear[Title/Abstract]) OR (Ear Acupunctures[Title/Abstract])) OR (Acupuncture, Auricular[Title/Abstract])) OR (Acupunctures, Auricular[Title/Abstract])) OR (Auricular Acupunctures[Title/Abstract])) OR (Auricular Acupuncture[Title/Abstract])) OR (Ear Acupuncture[Title/Abstract])))) OR (Moxibustion[Title/Abstract])** |
| #3 | #1 AND #2 |
| Filters： | (meta-analysis OR systematic review) |

**Embase: 102**

| Search | Query |
| --- | --- |
| #1 | 'pregnancy'/exp |
| #2 | 'pregnancies':ab,ti |
| #3 | 'gestation':ab,ti |
| #4 | #1 or #2 or #3 |
| #5 | 'nausea'/exp |
| #6 | 'antiemetics':ab,ti |
| #7 | 'emetics':ab,ti |
| #8 | #5 or #6 or #7 |
| #9 | 'vomiting'/exp |
| #10 | 'antiemetics':ab,ti |
| #11 | 'emetics':ab,ti |
| #12 | #9 or #10 or #11 |
| #13 | 'morning sickness'/exp |
| #14 | '**sickness, morning**':ab,ti |
| #15 | #13 or #14 |
| #16 | #8 or #12 or #15 |
| #17 | #4 and #16 |
| #18 | 'hyperemesis gravidarum'/exp |
| #19 | 'pernicious vomiting of pregnancy':ab,ti |
| #20 | 'pregnancy pernicious vomiting':ab,ti |
| #21 | #18 or #19 or #20 |
| #22 | #17 or #21 |
| #23 | 'acupuncture'/exp |
| #24 | 'pharmacopuncture':ab,ti |
| #25 | #23 or #24 |
| #26 | 'needle'/exp |
| #27 | 'needles':ab,ti |
| #28 | 'hypodermic needles':ab,ti |
| #29 | 'hypodermic needle':ab,ti |
| #30 | 'needle, hypodermic':ab,ti |
| #31 | 'needles, hypodermic':ab,ti |
| #32 | #26 or #27 or #28 or #29 or #30 or #31 |
| #33 | 'electroacupuncture'/exp |
| #34 | 'electro-acupuncture':ab,ti |
| #35 | #33 or #34 |
| #36 | #25 or #32 or #35 |
| #37 | #22 and #36 |

**Cochrane Central Register of Controlled Trials: 73**

| #1 | MeSH descriptor: [Pregnancy] explode all trees |
| --- | --- |
| #2 | (Pregnancies):ti,ab,kw OR (Gestation):ti,ab,kw |
| #3 | #1 OR #2 |
| #4 | MeSH descriptor: [Nausea] explode all trees |
| #5 | (Antiemetics):ti,ab,kw OR (Emetics):ti,ab,kw |
| #6 | #4 OR #5 |
| #7 | MeSH descriptor: [Vomiting] explode all trees |
| #8 | (Antiemetics):ti,ab,kw OR (Emetics):ti,ab,kw |
| #9 | #7 OR #8 |
| #10 | MeSH descriptor: [Morning Sickness] explode all trees |
| #11 | (Sickness, Morning):ti,ab,kw |
| #12 | #10 OR #11 |
| #13 | #6 OR #9 OR #12 |
| #14 | #3 AND #13 |
| #15 | MeSH descriptor: [Hyperemesis Gravidarum] explode all trees |
| #16 | (Pernicious Vomiting of Pregnancy):ti,ab,kw OR (Pregnancy Pernicious Vomiting):ti,ab,kw |
| #17 | #15 OR #16 |
| #18 | #14 OR #17 |
| #19 | MeSH descriptor: [Acupuncture] explode all trees |
| #20 | (Pharmacopuncture):ti,ab,kw |
| #21 | #19 OR #20 |
| #22 | MeSH descriptor: [Acupuncture Therapy] explode all trees |
| #23 | (Acupuncture Treatment):ti,ab,kw OR (Acupuncture Treatments):ti,ab,kw OR (Treatment, Acupuncture):ti,ab,kw OR (Therapy, Acupuncture):ti,ab,kw OR (Pharmacoacupuncture Treatment):ti,ab,kw OR (Treatment, Pharmacoacupuncture):ti,ab,kw OR (Pharmacoacupuncture Therapy):ti,ab,kw OR (Therapy, Pharmacoacupuncture):ti,ab,kw OR (Acupotomy):ti,ab,kw OR (Acupotomies):ti,ab,kw |
| #24 | #22 OR #23 |
| #25 | MeSH descriptor: [Needles] explode all trees |
| #26 | (Needle):ti,ab,kw OR (Needle, Hypodermic):ti,ab,kw OR (Hypodermic Needles):ti,ab,kw OR (Hypodermic Needle):ti,ab,kw OR (Needles, Hypodermic):ti,ab,kw |
| #27 | #25 OR #26 |
| #28 | MeSH descriptor: [Electroacupuncture] explode all trees |
| #29 | (Electro-acupuncture):ti,ab,kw |
| #30 | #28 OR #29 |
| #31 | MeSH descriptor: [Acupuncture, Ear] explode all trees |
| #32 | (Acupunctures, Auricular):ti,ab,kw OR (Acupuncture, Auricular):ti,ab,kw OR (Auricular Acupuncture):ti,ab,kw OR (Auricular Acupunctures):ti,ab,kw OR (Ear Acupunctures):ti,ab,kw OR (Auricular Acupunctures):ti,ab,kw OR (Acupunctures, Ear):ti,ab,kw |
| #33 | #31 OR #32 |
| #34 | #21 OR #24 OR #27 OR #30 OR #33 |
| #35 | #18 AND #34 |

**Web of Science: 40**

| #1 | TS = (**Pregnancy OR Pregnancies OR Gestation)** |
| --- | --- |
| #2 | TS = (**Nausea OR Vomiting OR Antiemetics OR Emetics OR Morning Sickness OR Sickness, Morning)** |
| #3 | #1 AND #2 |
| #4 | TS = (**Hyperemesis Gravidarum OR Pernicious Vomiting of Pregnancy OR Pregnancy Pernicious Vomiting)** |
| #5 | #3 OR #4 |
| #6 | TS = (**Acupuncture OR Pharmacopuncture OR** Acupuncture Therapy OR Acupuncture Treatment OR Acupuncture Treatments OR Treatment, Acupuncture OR Therapy, Acupuncture OR Pharmacoacupuncture Treatment OR Treatment, Pharmacoacupuncture OR Pharmacoacupuncture Therapy OR Therapy, Pharmacoacupuncture OR Acupotomy OR Acupotomies **OR Needles OR Hypodermic Needles OR Hypodermic Needle OR Needle, Hypodermic OR Needles, Hypodermic OR Electroacupuncture OR** Electro-acupuncture OR Moxibustion OR Acupoint application OR Acupuncture point injection OR Acupuncture, Ear OR Acupunctures, Ear OR Ear Acupunctures OR Acupuncture, Auricular OR Acupunctures, Auricular OR Auricular Acupunctures OR Auricular Acupuncture OR Ear Acupuncture**)** |
| #7 | #5 AND #6 |
| #8 | TS = (Meta Analysis OR Systematic Review) |
| #9 | #7 AND #8 |

**CINAHL: 11**

| #1 | ('pregnancy') OR XB ('pregnancies' OR 'gestation') |
| --- | --- |
| #2 | ('nausea') OR XB ('antiemetics' OR 'emetics') |
| #3 | ('vomiting') OR XB ('antiemetics' OR 'emetics') |
| #4 | ('morning sickness') OR XB ('sickness, morning') |
| #5 | ('hyperemesis gravidarum') OR XB ('pernicious vomiting of pregnancy' OR 'pregnancy pernicious vomiting') |
| #6 | (#2 OR #3 OR #4 OR #5) |
| #7 | #1 AND #6 |
| #8 | ('acupuncture therapy') OR XB ('acupuncture treatment' OR 'acupuncture treatments' OR 'treatment,acupuncture' OR 'therapy,acupuncture' OR 'pharmacoacupuncture treatment' OR 'treatment, pharmacoacupuncture' OR 'pharmacoacupuncture therapy' OR 'therapy, pharmacoacupuncture' OR 'acupotomy' OR 'acupotomies') |
| #9 | ('acupuncture analgesia') OR XB ('analgesia, acupuncture' OR 'acupuncture anesthesia' OR 'anesthesia, acupuncture') |
| #10 | ('acupuncture, ear') OR XB ('acupunctures, ear' OR 'ear acupunctures' OR 'acupuncture, auricular' OR 'acupunctures, auricular' OR 'auricular acupunctures' OR 'auricular acupuncture' OR 'ear acupuncture') |
| #11 | ('acupuncture points') OR XB ('acupuncture point' OR 'point, acupuncture' OR 'points, acupuncture' OR 'acupoints' OR 'acupoint') |
| #12 | ('needles') OR XB ('needle' OR 'hypodermic needles' OR 'hypodermic needle' OR 'needles,hypodermic' OR 'needle,hypodermic') |
| #13 | ('moxibustion') |
| #14 | ('electroacupuncture') |
| #15 | #8 OR #9 OR #10 OR #11 OR #12 OR #13 OR #14 |
| #16 | #7 AND #15 |
| #17 | ('Meta Analysis' OR 'Systematic Review') |
| #18 | #16 AND #17 |

**China National Knowledge Infrastructure: 5**

| #1 | SU='妊娠' |
| --- | --- |
| #2 | SU=('恶心'+'呕吐'+'恶阻'+'剧吐') |
| #3 | #1 AND #2 |
| #4 | SU=('针刺'+'干针'+'电针'+'耳针'+'耳穴'+'火针'+'手针'+'体针'+'毫针'+'温针'+'头针'+'腹针'+'腕踝针'+'眼针'+'水针'+'芒针'+'埋针'+'梅花针'+'艾灸'+'穴位注射'+'穴位贴敷') |
| #5 | #3 AND #4 |
| #6 | SU=( 'Meta分析'+'荟萃分析'+'系统评价'+'系统综述') |
| #7 | #5 AND #6 |

**Wanfang Database: 27**

| #1 | 主题:(妊娠) |
| --- | --- |
| #2 | 主题:(恶心 OR 呕吐 OR 恶阻 OR 剧吐) |
| #3 | #1 AND #2 |
| #4 | 主题:(针刺 OR 干针 OR 电针 OR 耳针 OR 耳穴 OR 火针 OR 手针 OR 体针 OR 毫针 OR 温针 OR 头针 OR 腹针 OR 腕踝针 OR 眼针 OR 水针 OR 芒针 OR 埋针 OR 梅花针 OR 艾灸OR穴位注射 OR穴位贴敷) |
| #5 | #3 AND #4 |
| #6 | 主题:(Meta分析 or 荟萃分析 or 系统评价 or 系统综述) |
| #7 | #5 AND #6 |

**Chinese Science and Technology Journals Database: 4**

| #1 | M=妊娠 |
| --- | --- |
| #2 | (M=恶心+M=呕吐+M=恶阻+M=剧吐) |
| #3 | #1 AND #2 |
| #4 | (M=针刺+M=干针+M=电针+M=耳针+M=耳穴+M=火针+M=手针+M=体针+M=毫针+M=温针+M=头针+M=腹针+M=腕踝针+M=眼针+M=水针+M=芒针+M=埋针+M= 梅花针+M= 艾灸+M= 穴位注射+M= 穴位贴敷) |
| #5 | #3 AND #4 |
| #6 | (U= Meta分析+U=荟萃分析+U=系统评价+U=系统综述) |
| #7 | #5 AND #6 |

**Chinese Biomedical Literature Database: 6**

| #1 | "恶心"[摘要:智能] OR "呕吐"[摘要:智能] OR "恶阻"[摘要:智能] OR "剧吐"[摘要:智能] |
| --- | --- |
| #2 | "妊娠"[摘要:智能] |
| #3 | #1 AND #2 |
| #4 | "针刺"[摘要:智能] OR "干针"[摘要:智能] OR "电针"[摘要:智能] OR "耳针"[摘要:智能] OR "耳穴"[摘要:智能] OR "火针"[摘要:智能] OR "手针"[摘要:智能] OR "体针"[摘要:智能] OR "毫针"[摘要:智能] OR "温针"[摘要:智能] OR "头针"[摘要:智能] OR "腹针"[摘要:智能] OR "腕踝针"[摘要:智能] OR "眼针"[摘要:智能] OR "水针"[摘要:智能] OR "芒针"[摘要:智能] OR "埋针"[摘要:智能] OR "梅花针"[摘要:智能] OR "艾灸"[摘要:智能] OR "穴位贴敷"[摘要:智能] OR "穴位注射"[摘要:智能] |
| #5 | #3 AND #4 |
| #6 | "Meta分析"[摘要:智能] OR "荟萃分析"[摘要:智能] OR "系统评价"[摘要:智能] OR "系统综述"[摘要:智能] |
| #7 | #5 AND #6 |
