# Supplementary material for: Acupuncture for nausea and vomiting during pregnancy: an overview of systematic reviews
Source: Front Endocrinol (Lausanne). 2026 Aug 7;17:1886667. doi: 10.3389/fendo.2026.1886667 (PMC13493242; doi:10.3389/fendo.2026.1886667)
Supplement: Supplementary file 3 [file SupplementaryFile2.docx]

Supplementary Material

# 1 Supplementary Figures

## 1.1 Supplementary Figure 1. Flow diagram of the study selection process.

## 1.2 Supplementary Figure 2. Evidence map of the methodological quality.

## 1.3 Supplementary Figure 3. Reporting quality assessment by PRISMA-A.

## 1.4 Supplementary Figure 4. Overall Reporting Quality Assessment of Each Study Included in the Systematic Review

## 1.5 Supplementary Figure 5. Matrix diagram of GROOVE
