# Supplementary material for: Acupuncture for nausea and vomiting during pregnancy: an overview of systematic reviews
Source: Front Endocrinol (Lausanne). 2026 Aug 7;17:1886667. doi: 10.3389/fendo.2026.1886667 (PMC13493242; doi:10.3389/fendo.2026.1886667)
Supplement: Supplementary file 4 [file Table1.docx]

Supplementary Material

# 1 Supplementary Tables

## 1.1 Supplementary Table 1. Basic characteristics of the included systematic reviews and meta-analyses.

| **Study** | **Country** | **Year** | **N/n** | **Intervention** | **Control** | **Assessment Tool** | **Outcome** | **Safety** | **Funding** |
| --- | --- | --- | --- | --- | --- | --- | --- | --- | --- |
| **Li** | China | 2017 | N = 11/n = 1043 | Acupuncture | Usual care | Cochrane risk of bias tool | ②③⑧⑭⑮ | Unknown | Yes |
| **Lin** | China | 2019 | N = 10/n = 834 | AIT **+** usual care | Usual care | Cochrane risk of bias tool; Modified Jadad score scale | ②⑧⑲ | Unknown | Yes |
| **Wan** | China | 2020 | N = 12/n = 1053 | AAT **+** usual care | Usual care | Cochrane risk of bias tool; Modified Jadad score scale | ⑧⑯⑰⑲ | Unknown | Unknown |
| **Lu** | China | 2021 | N = 16/n = 1043 | Acupuncture | Usual care; SA； | Cochrane risk of bias tool | ②③⑧⑪⑫⑬⑭⑮ | No | No |
| **Tian** | China | 2022 | N = 5/n = 359 | APA **+** usual care | Usual care | Modified Jadad score scale | ⑧⑲ | Unknown | Unknown |
| **Shu** | China | 2022 | N = 13/n = 1026 | APA | Usual care; SA; | Cochrane risk of bias tool | ①⑧ | Unknown | No |
| **Chen** | China | 2022 | N = 16/n = 1150 | AAT; AAT **+** TAU | Usual care | Cochrane risk of bias tool; Modified Jadad score scale | ②⑧⑪⑫⑳ | No | Unknown |
| **Jin** | China | 2024 | N = 24/n = 2390 | Acupuncture; Acupuncture **+** usual care | SA; usual care | Cochrane risk of bias tool | ①②③④⑤⑥⑦⑧⑨⑩ | Yes | Yes |
| **An** | China | 2025 | N = 35/n = 2672 | Acupuncture **+** usual care | Usual care | Cochrane risk of bias tool | ①⑧ | Unknown | Yes |
| Note: Acupuncture: Acupuncture; Acupoint application therapy: AAT; Auricular acupressure: APA; acupoint injection therapy: AIT; Sham acupuncture: SA; | | | | | | | | | |

## 1.2 Supplementary Table 2. Methodological quality assessment of the included systematic reviews

|  | **Li** | **Lin** | **Wan** | **Lu** | **Tian** | **Shu** | **Chen** | **Jin** | **An** |
| --- | --- | --- | --- | --- | --- | --- | --- | --- | --- |
| **Item 1** | Y | Y | Y | Y | Y | Y | Y | Y | Y |
| **Item 2** | N | N | N | Y | N | N | N | Y | Y |
| **Item 3** | N | N | N | N | N | N | N | N | N |
| **Item 4** | PY | PY | PY | Y | PY | PY | PY | Y | PY |
| **Item 5** | N | Y | N | Y | N | Y | Y | Y | Y |
| **Item 6** | N | Y | N | Y | N | Y | Y | Y | Y |
| **Item 7** | N | PY | PY | Y | PY | PY | PY | N | N |
| **Item 8** | PY | PY | PY | PY | PY | PY | PY | PY | PY |
| **Item 9** | Y | Y | N | Y | N | Y | PY | Y | Y |
| **Item 10** | N | N | N | N | N | N | N | N | N |
| **Item 11** | Y | Y | N | Y | N | N | Y | Y | Y |
| **Item 12** | N | N | N | Y | N | N | N | Y | N |
| **Item 13** | N | N | PY | Y | PY | Y | Y | Y | N |
| **Item 14** | Y | N | PY | Y | PY | Y | Y | Y | N |
| **Item 15** | N | Y | Y | Y | Y | N | Y | Y | Y |
| **Item 16** | N | N | Y | Y | Y | Y | N | Y | Y |
| **Ranking of quality** | **Critically low** | **Critically low** | **Critically low** | **Moderate** | **Critically low** | **Critically low** | **Low** | **Low** | **Critically low** |
| **AMSTAR: A Measurement Tool to Assess Systematic Reviews.** **N: no; PY: partial yes; Y: yes.** | | | | | | | | | |

## 1.3 Supplementary Table 3. Methodological quality assessment of the included systematic reviews

| **Study** | **Phase1** | **Phase2** | | | | **Phase3** |
| --- | --- | --- | --- | --- | --- | --- |
|  |  | **Study eligibility criteria** | **Identification and selection of studies** | **Data collection and study appraisal** | **Synthesis and findings** | **Risk of bias in the review** |
| **Li** | L | H | H | U | H | H |
| **Lin** | L | H | H | L | H | H |
| **Wan** | L | H | H | L | H | H |
| **Lu** | L | L | L | L | L | L |
| **Tian** | L | H | H | H | H | H |
| **Shu** | L | L | H | L | U | H |
| **Chen** | L | H | H | H | L | H |
| **Jin** | L | L | L | L | L | L |
| **An** | L | L | L | L | L | L |
| Note: L, low risk; H, high risk; U, unclear risk. | | | | | | |

## 1.4 Supplementary Table 4. Reporting quality assessment by PRISMA-A.

| **Item** | **Li** | **Lin** | **Wan** | **Lu** | **Tian** | **Shu** | **Chen** | **Jin** | **An** |
| --- | --- | --- | --- | --- | --- | --- | --- | --- | --- |
| **Item 1** | Y | Y | Y | Y | Y | Y | Y | Y | Y |
| **Item 2** | P | P | P | Y | P | P | P | Y | Y |
| **Item 3** | N | P | P | Y | P | P | P | P | Y |
| **Item 4** | Y | Y | Y | Y | Y | Y | Y | Y | Y |
| **Item 5** | N | N | N | Y | N | N | N | Y | Y |
| **Item 6** | P | P | P | P | P | P | P | P | P |
| **Item 7** | Y | P | P | Y | Y | Y | Y | Y | Y |
| **Item 8** | P | P | P | Y | P | P | P | Y | Y |
| **Item 9** | Y | Y | Y | Y | P | Y | Y | Y | Y |
| **Item 10** | Y | Y | Y | Y | P | Y | P | Y | Y |
| **Item 11** | Y | P | P | Y | P | N | P | Y | Y |
| **Item 12** | Y | Y | Y | Y | Y | Y | Y | Y | Y |
| **Item 13** | Y | Y | Y | Y | Y | Y | Y | Y | Y |
| **Item 14** | Y | Y | Y | Y | Y | Y | Y | Y | Y |
| **Item 15** | N | Y | Y | Y | Y | Y | Y | Y | Y |
| **Item 16** | N | Y | N | Y | N | N | N | Y | Y |
| **Item 17** | P | Y | Y | Y | Y | Y | Y | Y | Y |
| **Item 18** | P | P | P | P | P | P | P | P | P |
| **Item 19** | Y | Y | Y | Y | P | Y | P | Y | Y |
| **Item 20** | Y | Y | Y | Y | Y | Y | Y | Y | Y |
| **Item 21** | Y | Y | Y | Y | Y | N | Y | Y | Y |
| **Item 22** | N | Y | Y | Y | Y | N | Y | Y | Y |
| **Item 23** | N | Y | N | Y | N | N | N | Y | Y |
| **Item 24** | Y | Y | P | Y | P | Y | Y | Y | Y |
| **Item 25** | Y | Y | Y | Y | Y | Y | Y | Y | Y |
| **Item 26** | Y | Y | Y | Y | Y | Y | Y | Y | Y |
| **Item 27** | N | N | Y | Y | Y | Y | N | Y | N |
| **N: no; P: partial yes; Y: yes.** | | | | | | | | | |

## 1.5 Supplementary Table 5. Meta-analyses and GRADE evidence evaluation(Intervention: Acupuncture)

| **Outcome** | **Study** | **Intervention** | **Control** | **Included RCTs (participants)** | **Effect (95% CI)** | **Quality assessment** | | | | | **Quality of evidence** |
| --- | --- | --- | --- | --- | --- | --- | --- | --- | --- | --- | --- |
|  |  |  |  |  |  | **Risk of bias** | **Inconsistency** | **Indirectness** | **Imprecision** | **Publication bias** |  |
| **Overall response rate** | An | Acupuncture + Usual care | Usual care | 7(518) | RR=1.20 (1.01, 1.42) | -1^a^ | -1^b^ | 0 | 0 | -1^d^ | Very Low |
|  | Li | Acupuncture + ivgtt | Medications ＋ ivgtt | 2(104) | OR=7.03 (4.47, 11.06) | -1^a^ | 0 | 0 | 0 | -1^d^ | Low |
|  | Li | Acupuncture | ivgtt | 3(219) | OR=7.03 (4.47, 11.06) | -1^a^ | 0 | 0 | 0 | -1^d^ | Low |
|  | Lu | Acupuncture | Ivgtt; Medications | 2(113) | OR=8.11 (5.29, 12.43) | -1^a^ | 0 | 0 | 0 | -1^d^ | Low |
|  | Lu | Acupuncture + ivgtt; Acupuncture + medications | Ivgtt; Medications | 8(500) | OR=8.11 (5.29, 12.43) | -1^a^ | 0 | 0 | 0 | -1^d^ | Low |
| **Ineffective rates** | Jin | Acupuncture | Medications | 5(357) | RR=0.50 (0.30, 0.81) | -1^a^ | 0 | 0 | 0 | -1^d^ | Low |
| **PUQE** | Jin | Acupuncture + Medications | Medications | 6(523) | MD=-1.95 (-3.08, -0.81) | -1^a^ | -1^b^ | 0 | 0 | -1^d^ | Very Low |
|  | Jin | Acupuncture | Medications | 3(279) | MD=-0.80 (-3.06, 1.46) | -1^a^ | -1^b^ | 0 | -1^d^ | -1^d^ | Very Low |
|  | An | Acupuncture + Usual care | Usual care | 3(220) | MD=-2.67 (-4.25, -1.08) | -1^a^ | -1^b^ | 0 | 0 | -1^d^ | Very Low |
| **Anxiety** | Jin | Acupuncture + Medications | Medications | 2(246) | MD=-1.46 (-3.80, 0.87) | -1^a^ | -1^b^ | 0 | -1^d^ | -1^d^ | Very Low |
|  | Jin | Acupuncture | SA | 2(180) | MD=0.78 (-1.13, 2.69) | -1^a^ | 0 | 0 | -1^d^ | -1^d^ | Very Low |
| **Depression** | Jin | Acupuncture | SA | 2(246) | MD=-1.24 (-2.61, 0.13) | -1^a^ | 0 | 0 | -1^d^ | -1^d^ | Very Low |
|  | Jin | Acupuncture | SA | 2(180) | MD=0.20 (-2.05, 2.46) | -1^a^ | 0 | 0 | -1^d^ | -1^d^ | Very Low |
| **Ketonuria negative rates** | Jin | Acupuncture + Medications | Medications | 6(431) | RR=1.32 (1.14, 1.53) | -1^a^ | 0 | 0 | 0 | -1^d^ | Low |
|  | Jin | Acupuncture | Medications | 4(330) | RR=1.27 (0.85, 1.90) | -1^a^ | -1^b^ | 0 | -1^d^ | -1^d^ | Very Low |
|  | Lu | Acupuncture + Medications | Medications | 3(203) | RR=1.36 (1.15, 1.60) | -1^a^ | 0 | 0 | -1^d^ | -1^d^ | Very Low |
|  | Li | Acupuncture + ivgtt | Ivgtt | 1(40) | OR=4.85 (0.86, 27.22) | -1^a^ | -1^b^ | 0 | -1^d^ | -1^d^ | Very Low |
| **NVPQOL** | Jin | Acupuncture + Medications | Medications | 3(301) | MD=-10.89 (-13.92, -7.86) | -1^a^ | 0 | 0 | 0 | -1^d^ | Low |
| **length of stay (LOS)** | Jin | Acupuncture + Medications | Medications | 4(267) | MD=-2.89 (-4.01, -1.77) | -1^a^ | 0 | 0 | 0 | -1^d^ | Low |
|  | Lu | Acupuncture + Medications | Medications | 2(104) | MD=-3.78 (-5.39, -2.16) | -1^a^ | -1^b^ | 0 | 0 | -1^d^ | Very Low |
|  | Li | Acupuncture + ivgtt | Ivgtt | 2(104) | SMD=-2.32 (-3.01, -1.63) | -1^a^ | -1^b^ | 0 | 0 | -1^d^ | Very Low |
| **Chinese Medicine Syndrome Scale (CMS) score** | Jin | Acupuncture + Medications | Medications | 4(292) | SMD=-0.67 (-0.99, -0.36) | -1^a^ | 0 | 0 | 0 | -1^d^ | Low |
| **Adverse events** | Jin | Acupuncture | SA | 2(434) | RR=0.64 (0.28, 1.46) | -1^a^ | 0 | 0 | -1^d^ | -1^d^ | Very Low |
|  | Jin | Acupuncture | Medications | 2(236) | RR=0.67 (0.17, 2.69) | -1^a^ | 0 | 0 | -1^d^ | -1^d^ | Very Low |
| **Improvement rate of nausea and vomiting symptoms** | Lu | Acupuncture + Medications | Medications | 2(153) | OR=26.44 (3.54, 197.31) | -1^a^ | 0 | 0 | -1^d^ | -1^d^ | Very Low |
| **Improvement in fatigue** | Lu | Acupuncture + Medications | Medications | 2(122) | RR=1.01 (0.89, 1.14) | -1^a^ | 0 | 0 | -1^d^ | -1^d^ | Very Low |
| **Pregnancy termination rate** | Li | Acupuncture + ivgtt | Ivgtt | 1(40) | OR=0.14 (0.02, 0.75) | -1^a^ | -1^b^ | 0 | -1^d^ | -1^d^ | Very Low |
|  | Lu | Acupuncture + Medications | Medications | 1(40) | RR=0.26 (0.09, 0.74) | -1^a^ | 0 | 0 | -1^d^ | -1^d^ | Very Low |
|  | Lu | Acupuncture | Medications | 1(95) | RR=0.26 (0.09, 0.74) | -1^a^ | 0 | 0 | -1^d^ | -1^d^ | Very Low |
| **Serum potassium** | Li | Acupuncture + ivgtt | Ivgtt | 1(40) | MD=0.11 (-0.04, 0.25) | -1^a^ | 0 | 0 | -1^d^ | -1^d^ | Very Low |
|  | Li | Acupuncture | Ivgtt | 1(95) | MD=0.11 (-0.04, 0.25) | -1^a^ | 0 | 0 | -1^d^ | -1^d^ | Very Low |
|  | Lu | Acupuncture | Medications | 1(95) | MD=0.11 (-0.04, 0.25) | -1^a^ | 0 | 0 | -1^d^ | -1^d^ | Very Low |
|  | Lu | Acupuncture + Medications | Medications | 1(40) | MD=0.11 (-0.04, 0.25) | -1^a^ | 0 | 0 | -1^d^ | -1^d^ | Very Low |
| **Improvement in dietary intake/food consumption** | Lu | Acupuncture | Medications | 1(81) | MD=0.11 (-0.04, 0.25) | -1^a^ | 0 | 0 | -1^d^ | -1^d^ | Very Low |
|  | Lu | Acupuncture + Medications | Medications | 2(166) | MD=0.11 (-0.04, 0.25) | -1^a^ | 0 | 0 | -1^d^ | -1^d^ | Very Low |
| Note: RR, relative risk; SMD, standardized mean difference; OR, odds ratio; MD, mean difference. a: The original studies had methodological problems related to blinding, randomization, allocation concealment, or other aspects of study design. b: Substantial heterogeneity was observed among the included studies. c: The included studies had small sample sizes and wide confidence intervals. d: Publication bias was possible, substantial publication bias was mentioned in the article, the funnel plot was asymmetric, or publication bias was not assessed.  Acupuncture: Acupuncture; Intravenous fluid therapy: ivgtt; Sham acupuncture: SA; | | | | | | | | | | | |

## 1.6 Supplementary Table 6 Meta-analyses and GRADE evidence evaluation(Intervention: Acupuncture and Moxibustion)

| **Outcome** | **Study** | **Intervention** | **Control** | **Included RCTs (participants)** | **Effect (95% CI)** | **Quality assessment** | | | | | **Quality of evidence** |
| --- | --- | --- | --- | --- | --- | --- | --- | --- | --- | --- | --- |
|  |  |  |  |  |  | **Risk of bias** | **Inconsistency** | **Indirectness** | **Imprecision** | **Publication bias** |  |
| **Overall response rate** | Lu | AMT | Medications | 1(100) | OR=8.11 (5.29, 12.43) | -1^a^ | 0 | 0 | 0 | -1^d^ | Low |
|  | Lu | AMT＋ ivgtt | Ivgtt | 1(70) | OR=8.11 (5.29, 12.43) | -1^a^ | 0 | 0 | 0 | -1^d^ | Low |
|  | Li | AMT | Ivgtt | 1(70) | OR=7.03 (4.47, 11.06) | -1^a^ | 0 | 0 | 0 | -1^d^ | Low |
| OR, odds ratio;  Intravenous fluid therapy: ivgtt; Acupuncture and moxibustion therapy: AMT; | | | | | | | | | | | |

## 1.7 Supplementary Table 7 Meta-analyses and GRADE evidence evaluation(Intervention: Acupuncture modalities)

| **Outcome** | **Study** | **Intervention** | **Control** | **Included RCTs (participants)** | **Effect (95% CI)** | **Quality assessment** | | | | | **Quality of evidence** |
| --- | --- | --- | --- | --- | --- | --- | --- | --- | --- | --- | --- |
|  |  |  |  |  |  | **Risk of bias** | **Inconsistency** | **Indirectness** | **Imprecision** | **Publication bias** |  |
| **Overall response rate** | An | MWM + Usual care | Usual care | 2(175) | RR=1.41 (1.14, 1.73) | -1^a^ | 0 | 0 | 0 | -1^d^ | Low |
|  | An | AIT + Usual care | Usual care | 3(269) | RR=1.20 (1.09, 1.32) | -1^a^ | 0 | 0 | 0 | -1^d^ | Low |
|  | An | AAT + Usual care | Usual care | 13(919) | RR=1.19 (1.13, 1.25) | -1^a^ | 0 | 0 | 0 | -1^d^ | Low |
|  | Tian | APA **+** Usual care | Usual care | 5(359) | OR=5.01 (2.50, 10.07) | -1^a^ | 0 | 0 | 0 | -1^d^ | Low |
|  | Lin | AIT **+** ivgtt | Ivgtt | 7(524) | OR=4.95 (2.66, 9.23) | -1^a^ | 0 | 0 | 0 | -1^d^ | Low |
|  | Lin | AIT | Ivgtt | 3(310) | OR=4.95 (2.66, 9.23) | -1^a^ | 0 | 0 | 0 | -1^d^ | Low |
|  | Chen | AAT **+** Ivgtt | Ivgtt | 8(596) | OR=4.66 (3.08, 7.03) | -1^a^ | 0 | 0 | 0 | 0 | Moderate |
|  | Chen | AAT | Ivgtt | 4(240) | OR=4.66 (3.08, 7.03) | -1^a^ | 0 | 0 | 0 | 0 | Moderate |
|  | Wan | AAT **+** ivgtt | Ivgtt | 6(424) | OR=7.49(4.64, 12.09) | -1^a^ | 0 | 0 | 0 | -1^d^ | Low |
|  | Wan | AAT **+** TCM **+** ivgtt | Ivgtt **+** TCM | 3(284) | OR=7.49(4.64, 12.09) | -1^a^ | 0 | 0 | 0 | -1^d^ | Low |
| **Cure rate** | Tian | AAT **+** Usual care | Usual care | 5(359) | OR=2.42 (2.14, 5.45) | -1^a^ | 0 | 0 | 0 | -1^d^ | Low |
|  | Lin | AIT **+ i**vgtt | Ivgtt | 7(524) | OR=4.18 (3.02, 5.78) | -1^a^ | 0 | 0 | 0 | 0 | Moderate |
|  | Lin | AIT | Ivgtt | 3(310) | OR=4.18 (3.02, 5.78) | -1^a^ | 0 | 0 | 0 | 0 | Moderate |
|  | Wan | AAT **+** ivgtt | Ivgtt | 2(272) | OR=3.25 (2.93, 4.52) | -1^a^ | 0 | 0 | 0 | -1^d^ | Low |
|  | Wan | AAT **+** TCM **+** ivgtt | Usual care **+** TCM | 1(102) | OR=3.25 (2.93, 4.52) | -1^a^ | 0 | 0 | 0 | -1^d^ | Low |
| **PUQE** | An | AIT + Usual care | Usual care | 4(352) | MD=-2.30 (-2.82, -1.79) | -1^a^ | -1^b^ | 0 | 0 | -1^d^ | Very Low |
|  | An | AAT + Usual care | Usual care | 3(224) | MD=-1.06 (-1.77, -0.35) | -1^a^ | -1^b^ | 0 | 0 | -1^d^ | Very Low |
| **Recurrence rate**  **Ketonuria negative rates** | Chen | AAT **+ i**vgtt | Ivgtt | 4(375) | OR=0.14 (0.08, 0.24) | -1^a^ | 0 | 0 | 0 | -1^d^ | Low |
|  | Lin | AIT **+** ivgtt | Ivgtt | 6(428) | OR=7.51 (4.05, 13.90) | -1^a^ | 0 | 0 | 0 | -1^d^ | Low |
|  | Lin | AIT | Ivgtt | 2(210) | OR=7.51 (4.05, 13.90) | -1^a^ | 0 | 0 | 0 | -1^d^ | Low |
|  | Chen | AAT **+** ivgtt | Ivgtt | 2(186) | OR=3.78 (1.86, 7.67) | -1^a^ | 0 | 0 | 0 | -1^d^ | Low |
|  | Chen | AAT | Ivgtt | 1(60) | OR=3.78 (1.86, 7.67) | -1^a^ | 0 | 0 | 0 | -1^d^ | Low |
| **Improvement rate of nausea and vomiting symptoms** | Shu | APA | SA | 1(128) | P<0.001 | -1^a^ | -1^b^ | 0 | -1^d^ | -1^d^ | Very Low |
|  | Shu | APA＋ivgtt | Ivgtt | 9(646) | P<0.001 | -1^a^ | -1^b^ | 0 | -1^d^ | -1^d^ | Very Low |
|  | Shu | APA | Ivgtt | 2(154) | P<0.001 | -1^a^ | -1^b^ | 0 | -1^d^ | -1^d^ | Very Low |
| Note: RR, relative risk; SMD, standardized mean difference; OR, odds ratio; MD, mean difference. a: The original studies had methodological problems related to blinding, randomization, allocation concealment, or other aspects of study design. b: Substantial heterogeneity was observed among the included studies. c: The included studies had small sample sizes and wide confidence intervals. d: Publication bias was possible, substantial publication bias was mentioned in the article, the funnel plot was asymmetric, or publication bias was not assessed.  Intravenous fluid therapy: ivgtt; Acupoint application therapy: AAT; Auricular acupressure: APA; acupoint injection therapy: AIT; Sham acupuncture: SA; Traditional Chinese medicine: TCM; Warm needle moxibustion: MWM; | | | | | | | | | | | |
